# Supplementary material for: The HER2-directed antibody-drug conjugate DHES0815A in advanced and/or metastatic breast cancer: preclinical characterization and phase 1 trial results
Source: Nat Commun. 2024 Jan 11;15:466. doi: 10.1038/s41467-023-44533-z (PMC10784567; doi:10.1038/s41467-023-44533-z)
Supplement: Supplementary file 1 — Supplementary Information [file 41467_2023_44533_MOESM1_ESM.pdf]

Supplementary Fig. 1

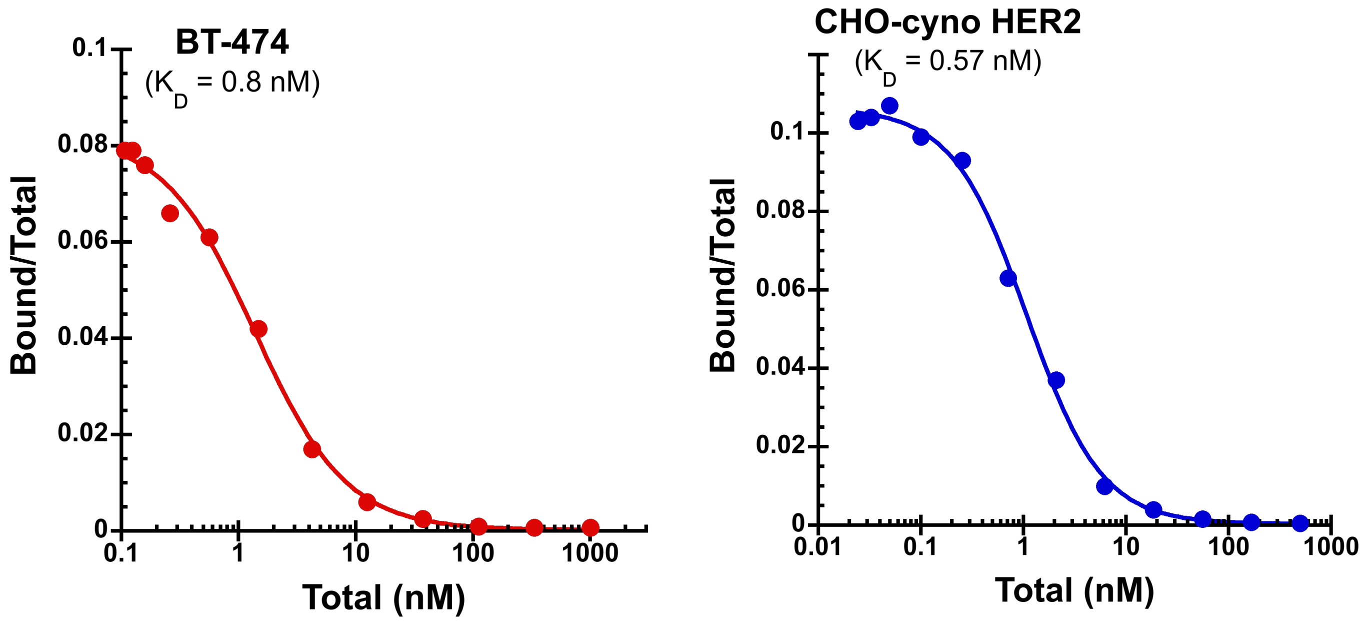

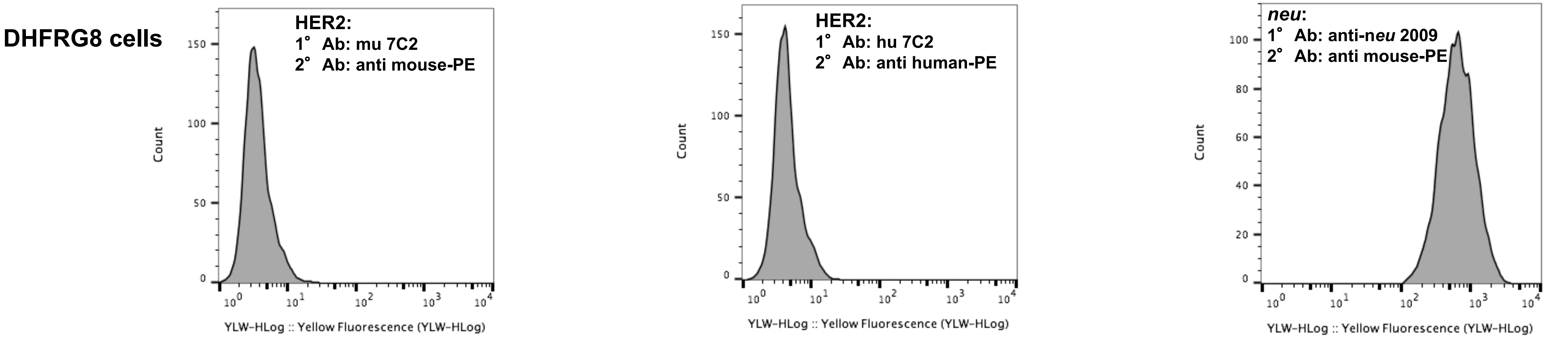

Supplementary Fig. 1. Hu7C2 binding to human and cynomolgus monkey HER2; hu7C2 does not bind rat neu. Top panels: Scatchard analysis using radiolabeled (^{125}I) hu7C2 was performed on BT-474 and CHO-cyno HER2 cells (CHO-cyno HER2 are Chines Hamster Ovary cells engineered to express cynomolgus monkey HER2). Bottom panels: FACS analysis on DHFRG8 cells (which overexpress rodent *neu)* showing no binding of murine or human 7C2 to rodent neu. Anti-neu Mab 2009 (1) shows strong binding to DHFRG8 cells. Source data are provided as a Source Data file.

Supplementary Fig. 2

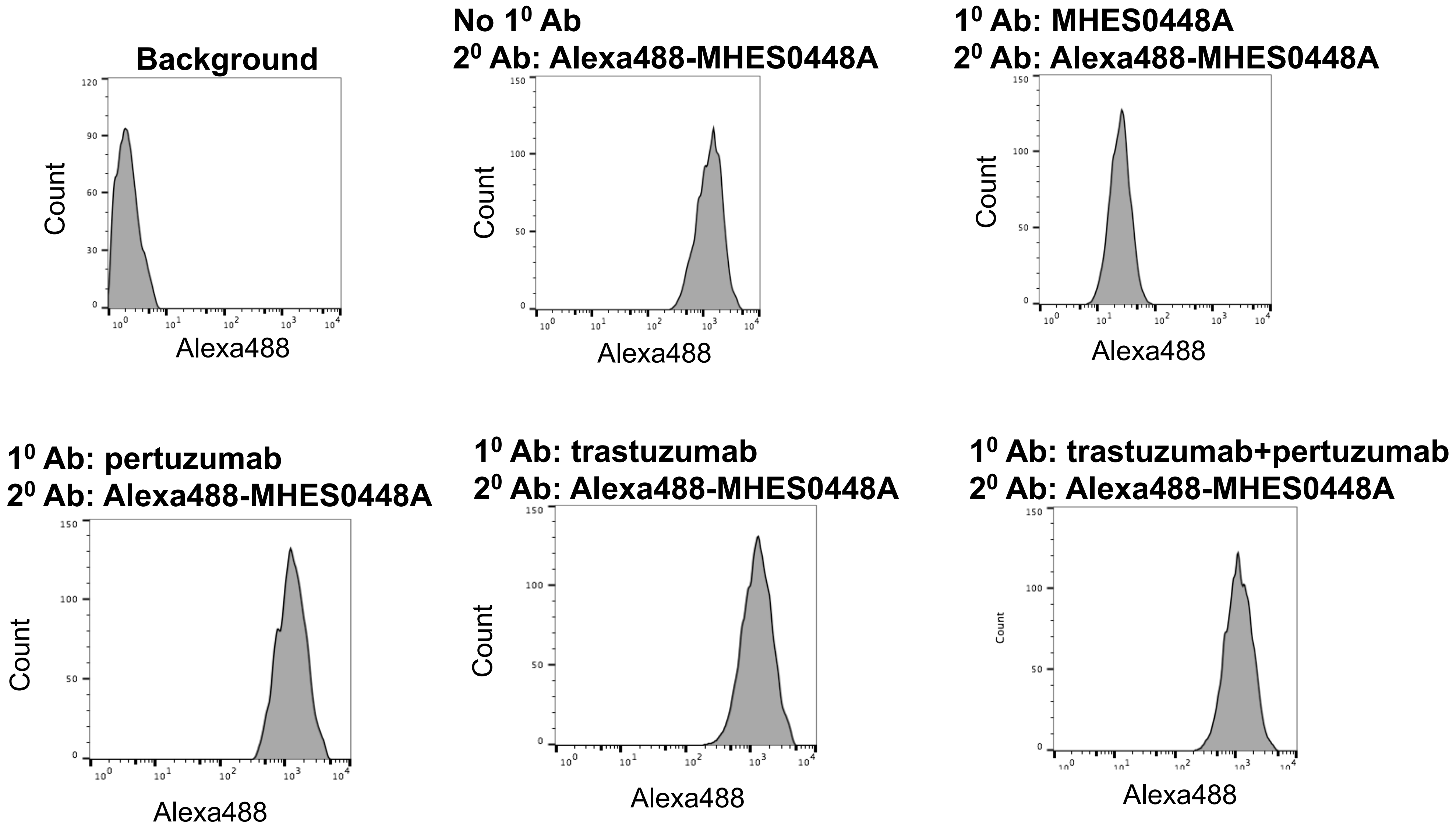

Supplementary Fig. 2. FACS analysis for MHES0448A binding in the presence of trastuzumab and/or pertuzumab in SK-BR-3 cells
To confirm that MHES0448A did not compete for binding with trastuzumab, pertuzumab or the combination, cells were treated with single, double or triple combination antibodies and binding fluorescence measured using Alexa488 labeled MHES0448A.

Supplementary Fig. 3

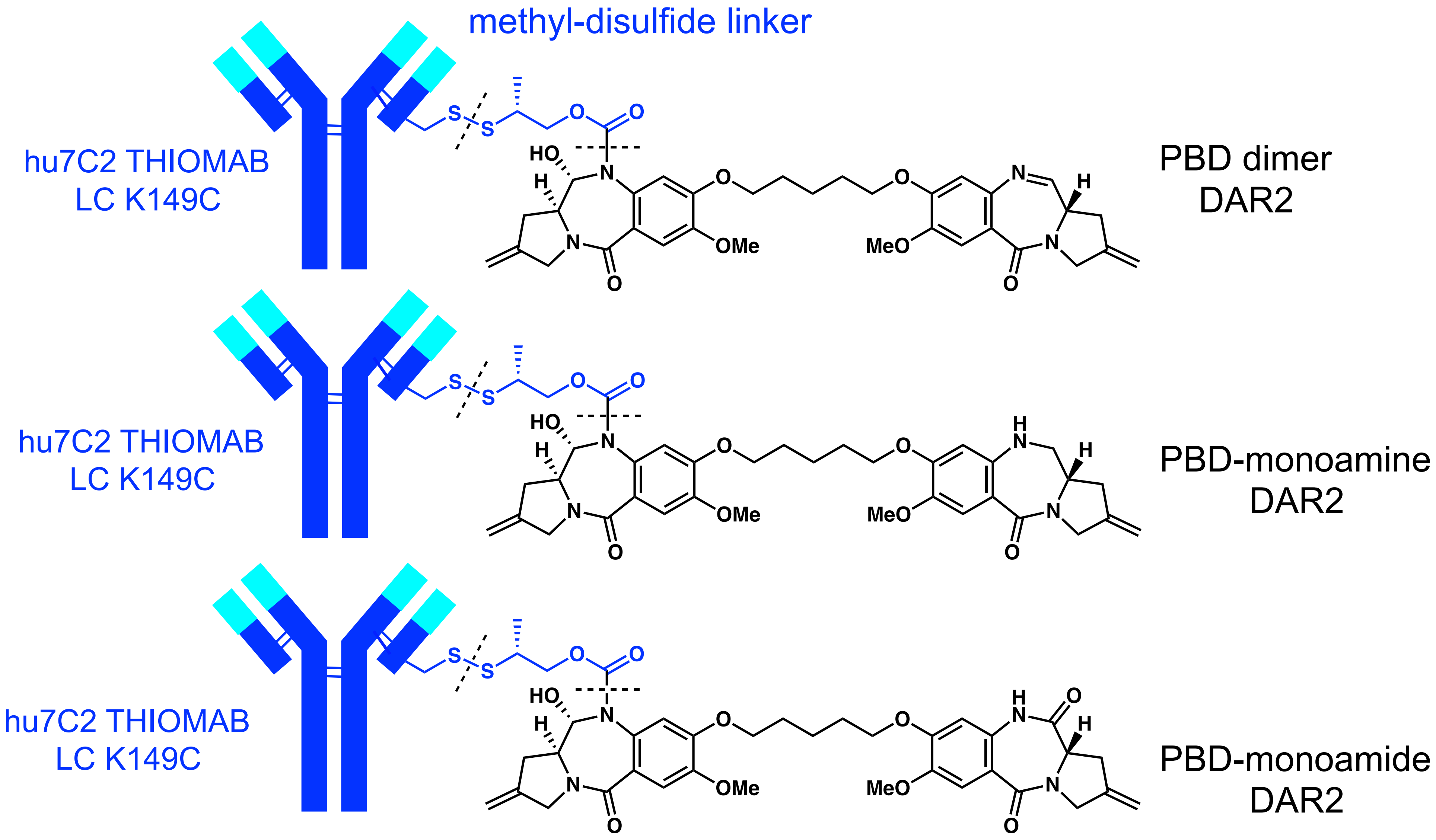

Supplementary Fig. 3. Structures of PBD dimer, PBD-monoamine and PBD-monoamide conjugated to hu7C2 THIOMAB LC K149C antibody with the methyl-disulfide linker.

Supplementary Fig. 4

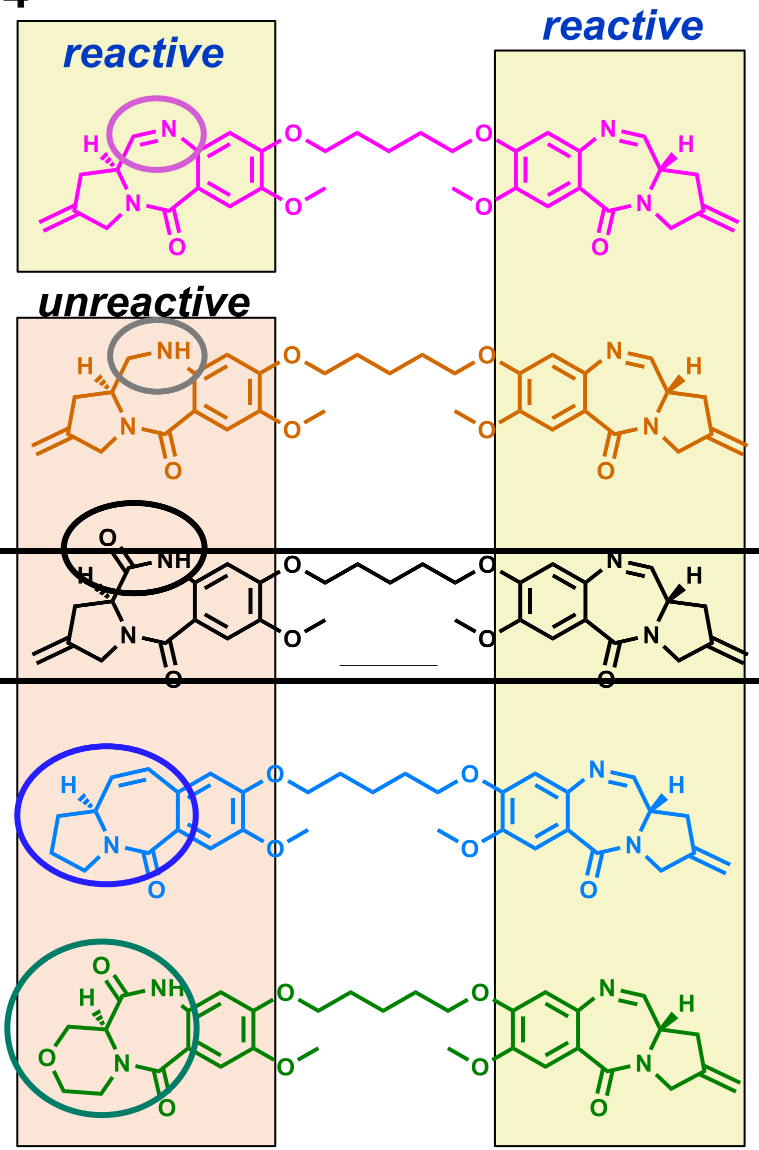

	Ave IC_{50}, free drugs (nM, 8-9 cell lines)	DNA binding (% alkylation)	IC_{50}, ADC (SK-BR-3)	MED in vivo
PBD dimer	1.6 ± 0.6	99%	2.9 ng/mL	1 mg/kg
monoamine	8.6 ± 3.0	93%	4.7 ng/mL	~2 mg/kg
monoamide	27.1 ± 2.0	58%	21.7 ng/mL	6-12 mg/kg
olefin	34.8 ±0.3	84%	Inactive	Inactive
morpholine	88.4 ± 0.9	79%	4 ng/mL	<6 mg/kg

Supplementary Fig. 4. Characterization of mono-alkylator derivatives of the PBD dimer
The parent PBD dimer was chemically modified on one half to convert the reactive imine to a DNA-unreactive moiety, yielding mono-alkylating PBDs. The derivatives were assessed for in vitro potency as free drugs and ADCs, DNA binding and in vivo efficacy. Free drug assays were carried out in large cell line screens (7 screens for PBD; 3 screens for monoamide; and 2 screens for monoamine, olefin and morpholine derivatives). See Methods section for details.

Supplementary Fig. 5

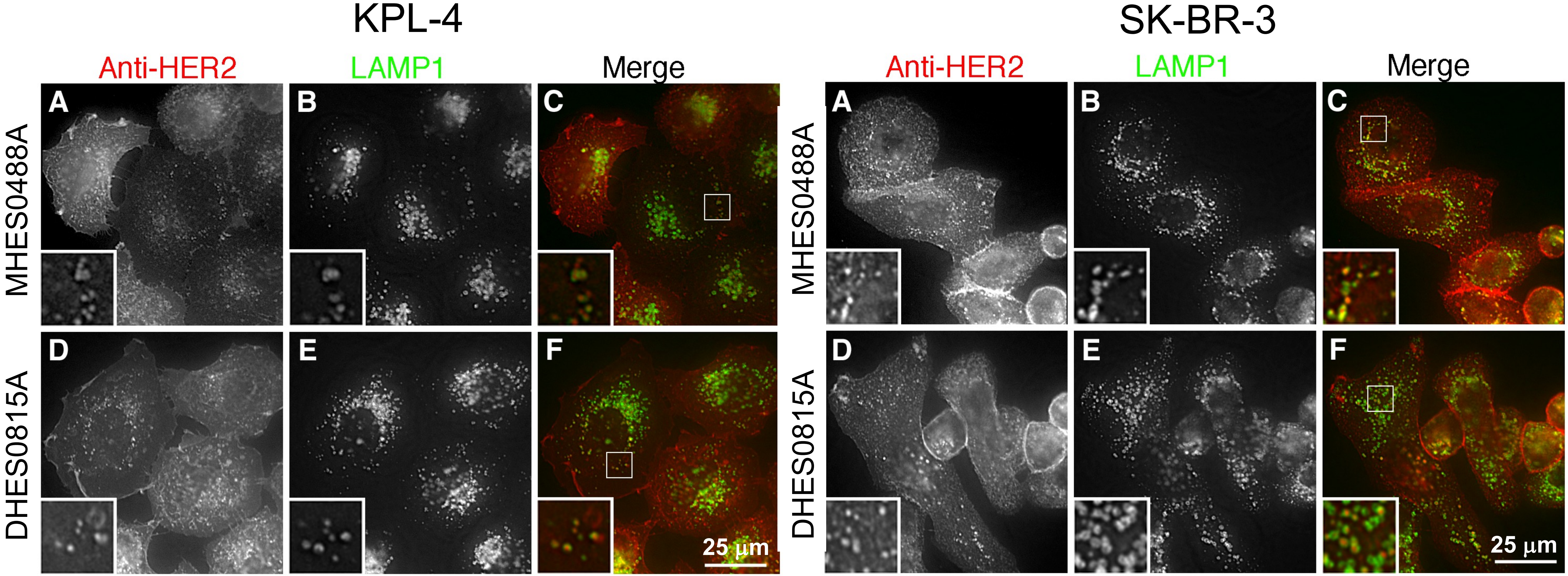

Supplementary Fig. 5. Internalization of DHES0815A into HER2-positive breast cancer cells
To determine whether MHES0488A and DHES0815A were internalized upon binding, HER2-over-expressing breast cancer cells were incubated overnight with MHES0488A or DHES0815A. Binding was detected with Cy3-conjugated anti-human secondary antibodies. MHES0448A was internalized into KPL-4 and SK-BR-3 cells (panels A-C), and was detected inside LAMP1-positive late endosomes and lysosomes. DHES0815A was similarly accumulated in lysosomes (panels D-F), indicating that conjugation of the PDB monoamide does not affect MHES0488 uptake or trafficking. Scale bar represents 25 micron; magnification is 600X (insets are 3X magnification of image). Experiment was repeated 2 times with similar results.

MHES0488A: huTHIO-7C2 LC K149C (naked antibody); DHES0815A: huTHIO-7C2 LC K149C disulfide-PBD-monoamide (conjugated antibody)

Supplementary Fig. 6

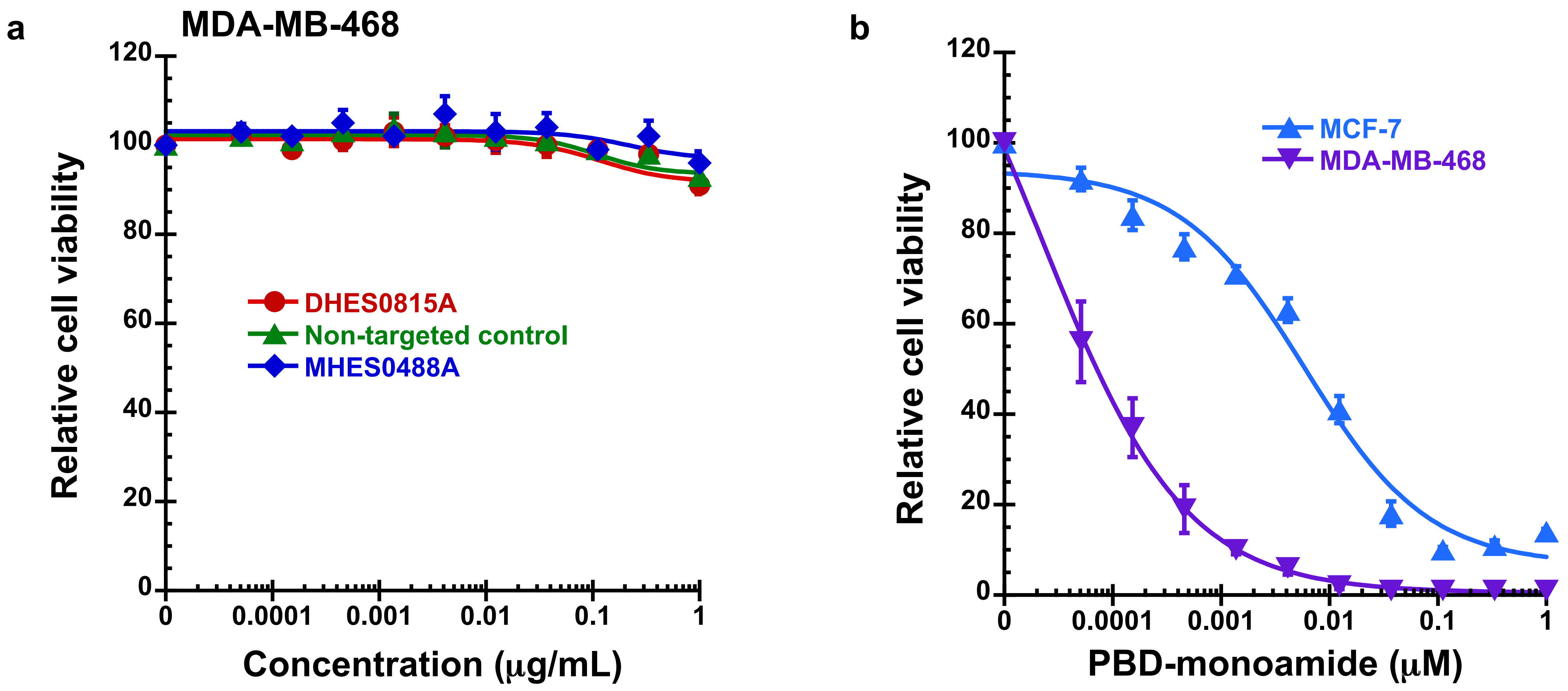

Supplementary Fig. 6. MDA-MB-468 HER2-negative breast cancer cells do not respond to HER2- or control antibody-conjugated PBD-monoamide (a); response of HER2-negative MDA-MB-468 and MCF7 to unconjugated PBD-monoamide (b). HER2-negative breast cancer cells were treated for 5 days with conjugated PBD-monoamide (DHES0815A vs. non-targeted control antibody conjugated to PBD-monoamide) or to unconjugated antibody MHES0488A, or for 4 days with different concentrations of PBD-monoamide. Cell viability was assessed using Cell Titer-Glo. Treatment groups were n=4 per experiment; data are mean ± standard error of the mean for 3 pooled independent experiments. Source data are provided as a Source Data file.

Supplementary Fig. 7

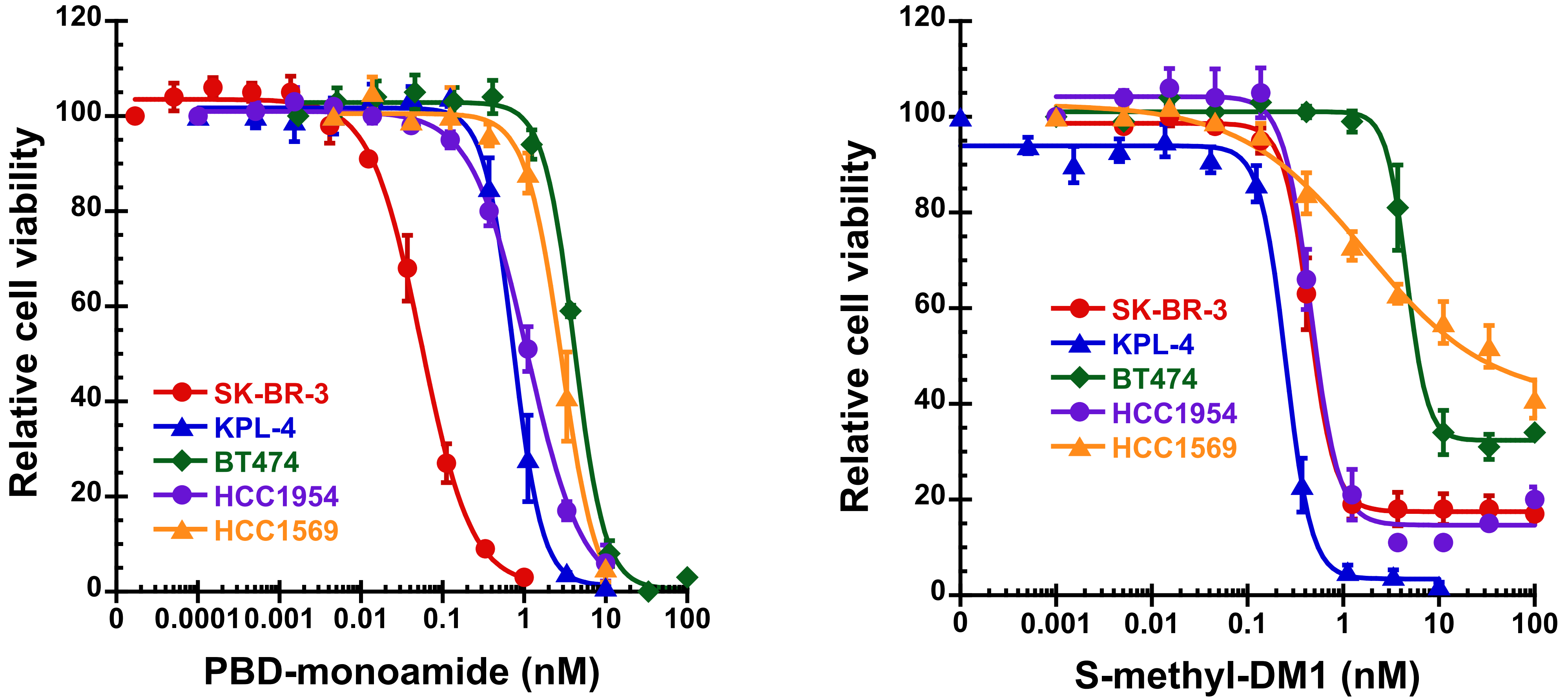

Supplementary Fig. 7. Breast cancer cell line response to PBD-monoamide vs. S-methyl DM1.
HER2+ breast cancer cells were treated for 4 days with different concentrations of PBD-monoamide or S-methyl-DM1 (cell permeable form of DM1). Cell viability was assessed using Cell Titer-Glo. Treatment groups were n=4 wells per treatment; data are mean± standard error of the mean for 3 pooled independent experiments. Source data are provided as a Source Data file.

Supplementary Fig. 8

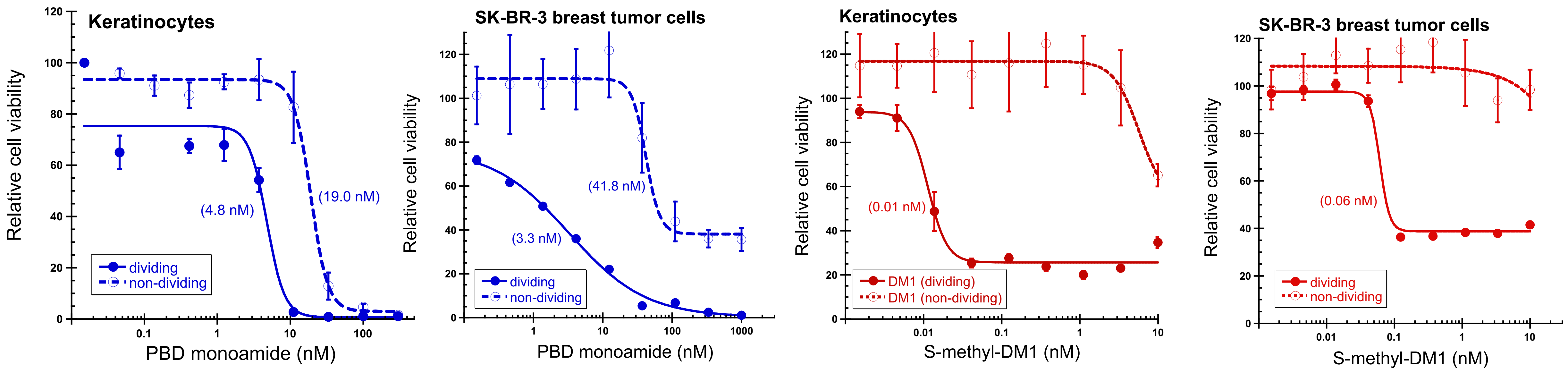

Supplementary Fig. 8. PBD monoamide decreases viability of dividing and non-dividing cells. Proliferating and quiescent human keratinocytes and SK-BR-3 breast cancer cells were treated with either PBDma or DM1 to assess changes in cell viability. Cell viability was reduced by PBD-monoamide in both dividing (proliferating) and non-dividing (quiescent) cells. In contrast, treatment with DM1 resulted in decreased cell viability only in actively dividing cells. Keratinocytes were growth-arrested by culturing the cells to confluence to induce contact inhibition. SK-BR-3 cells were cultured in serum-free medium to induce quiescence. Treatment groups were n=3 wells (96-well plate) per group; data are represented as mean ± standard error (s.e.). Source data are provided as a Source Data file.

Supplementary Fig. 9

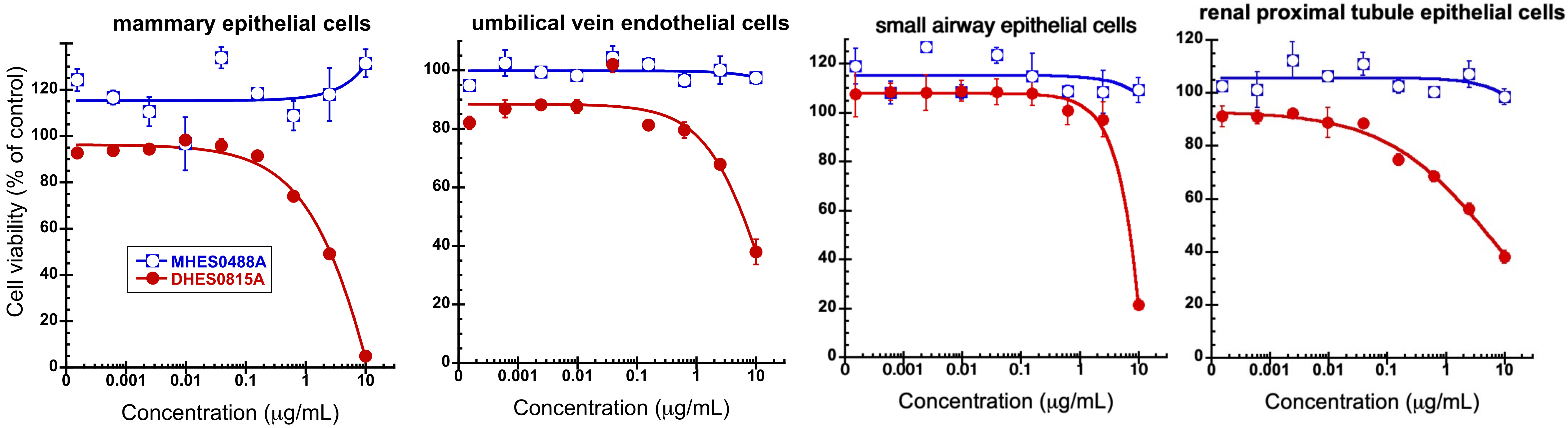

Supplementary Fig. 9. DHES0815A has minimal effect on proliferation of normal cells

Exposure of normal human cells (normal human keratinocytes, human mammary epithelial cells, human umbilical vein endothelial cells, human renal proximal tubule epithelial cells, and human small airway epithelial cells) to DHES0815A resulted in minimal growth inhibition. 7C2 (MHES0488A) treatment had no effect on viability of normal cells. Treatment groups were n=3 wells (of 96-well plate) per group; data are represented as mean ± standard error (s.e.). Source data are provided as a Source Data file.

Supplementary Fig. 10

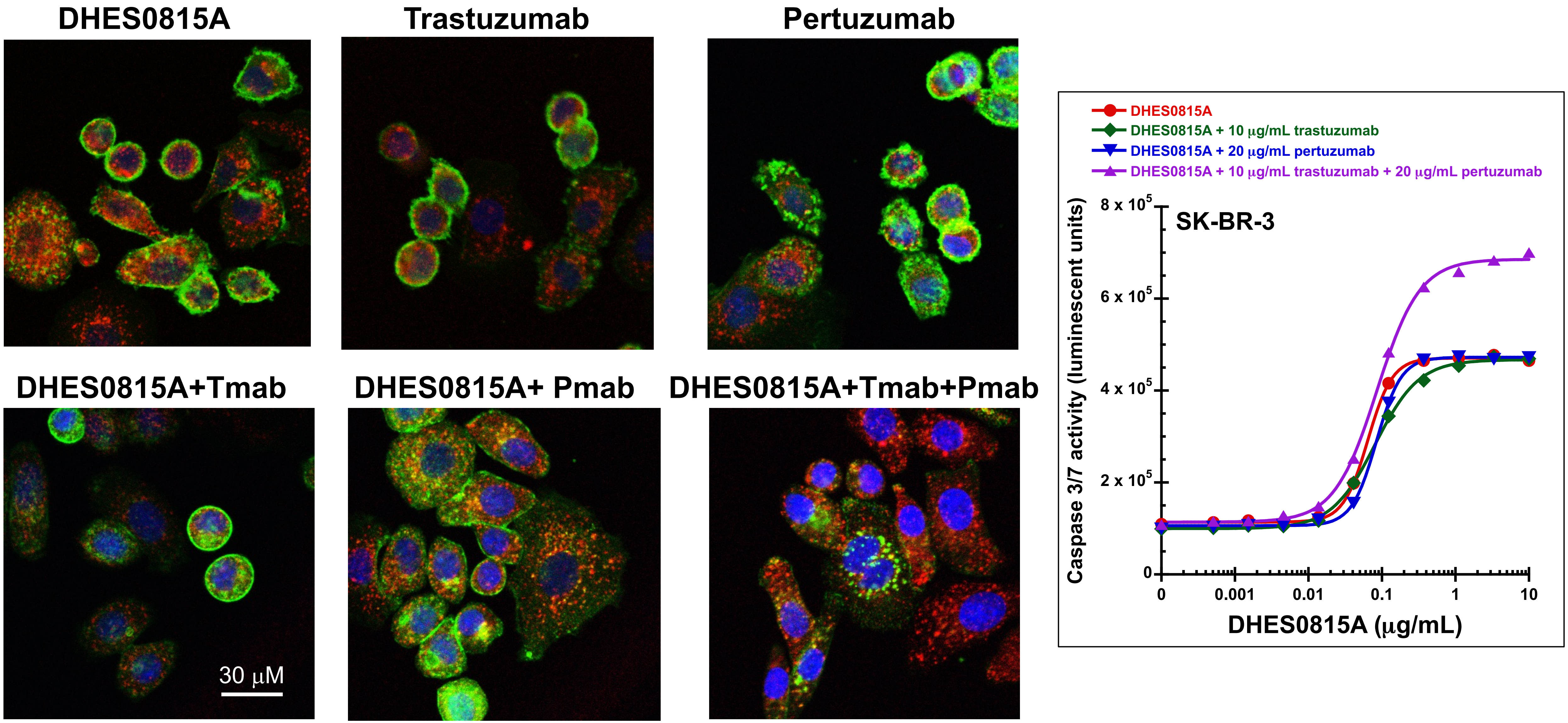

Supplementary Fig. 11. Enhanced HER2 internalization and apoptosis with DHES0815A combined with trastuzumab and pertuzumab. HER2 internalization, indicated by disappearance of green fluorescent signal from the cell membrane, was strongly enhanced with the combination of DHES0815A, trastuzumab (Tmab) and pertuzumab (Pmab) compared to single or double agent treatment (study repeated 3 times with similar results. Apoptosis was similarly enhanced with the triple combination vs. DHES0815A alone or combined with either trastuzumab or pertuzumab (n=4 per group, data are mean values from 2 independent experiments). Source data are provided as a Source Data file.

Supplementary Fig. 11

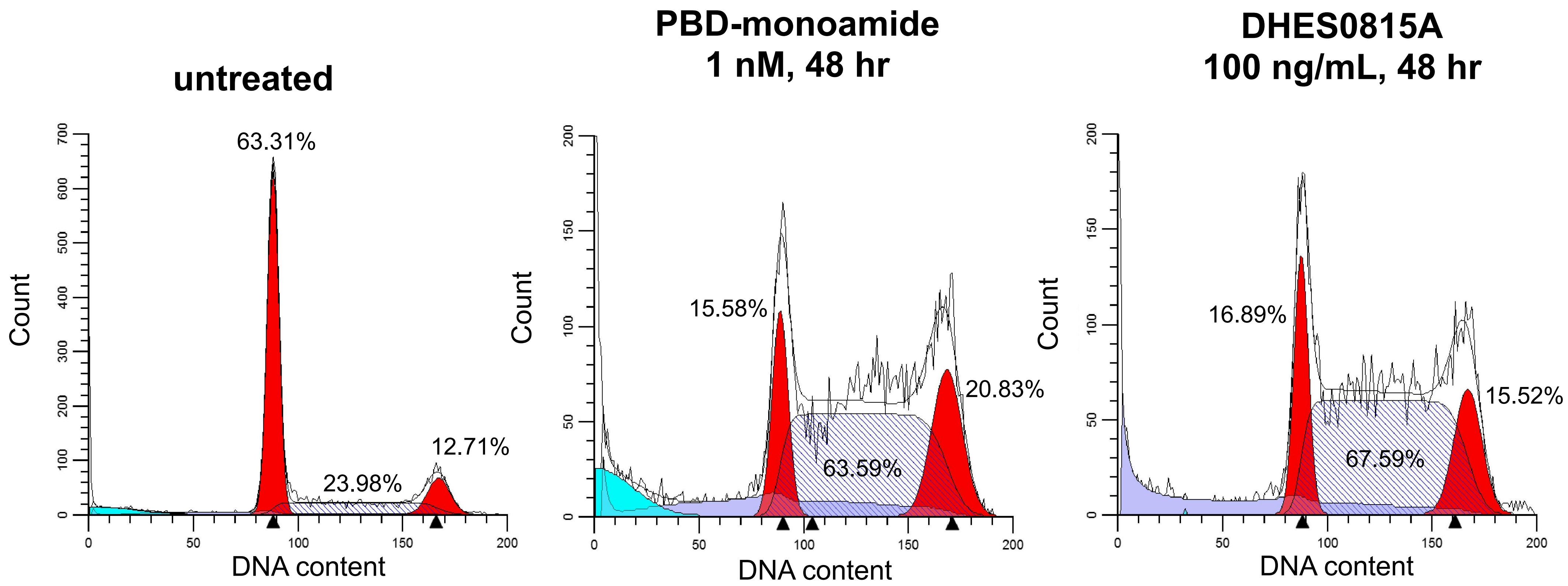

Supplementary Fig. 12. Effect of DHES0815A and PBD-monoamide on cell cycle progression in SK-BR-3 cells.
Cells were incubated for 48 hr with free PBD-monoamide or DHES0815A, harvested and DNA stained with propidium iodide for cell cycle analysis.
Numbers represent percent cells in each cell cycle phase (G1, S, G2/M).

Supplementary Fig. 12

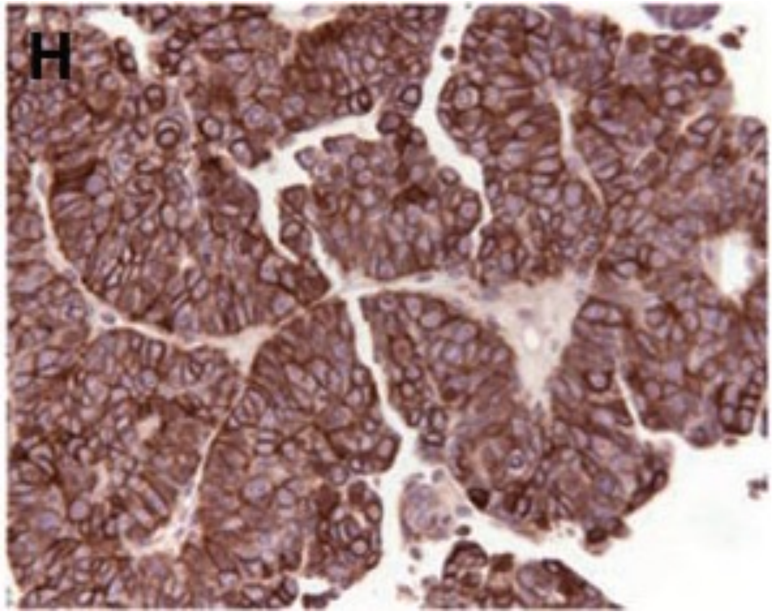

MMTV-HER2 fo5 transgenic tumor; HER2 IHC 3+
HER2 IHC (Finkle et al., Clin Cancer Res 2004)

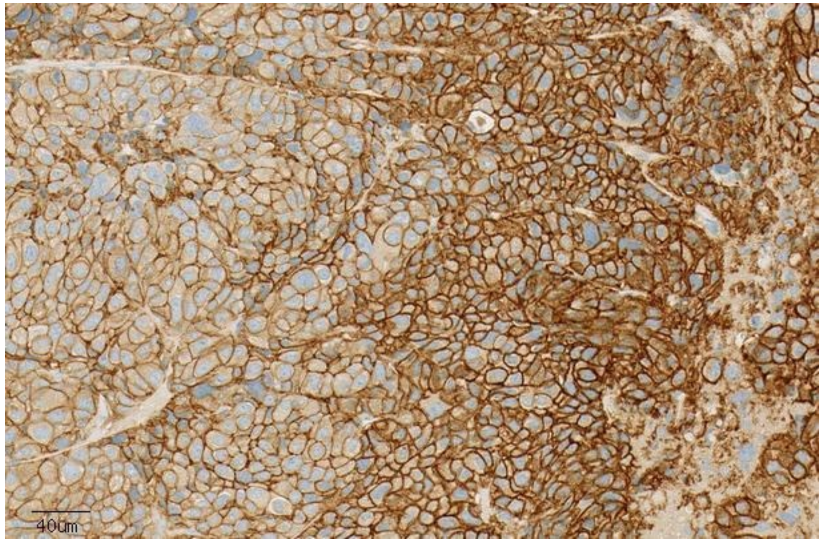

WHIM8 PDX model; HER2 IHC 3+

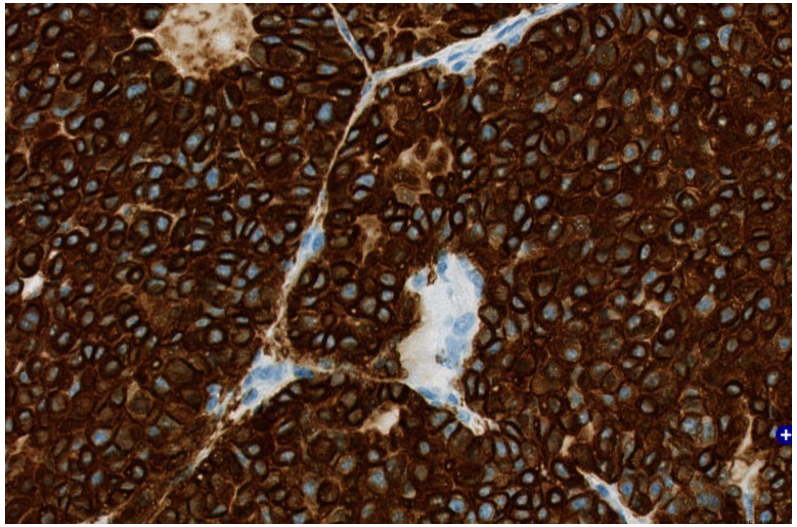

MMTV-HER2 fo5 transgenic tumor fragment; HER2 IHC 3+
(used for xenograft studies)

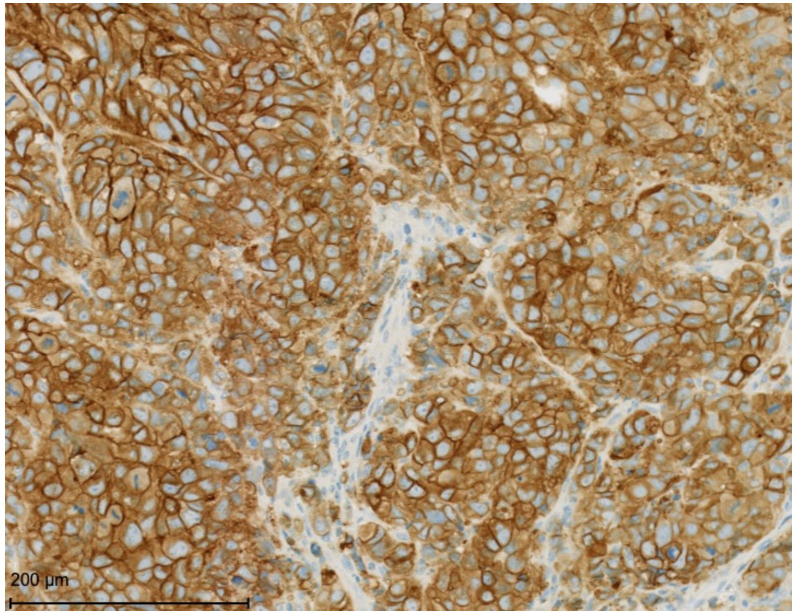

HCC1569 X2 breast cancer cell line model; HER2 IHC 3+

Supplementary Fig. 12, continued

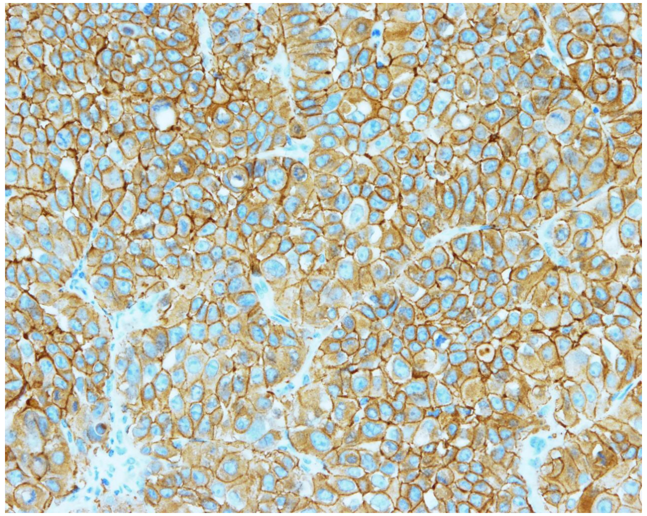

STO41 gastric cancer PDX model
HER2 IHC 3+

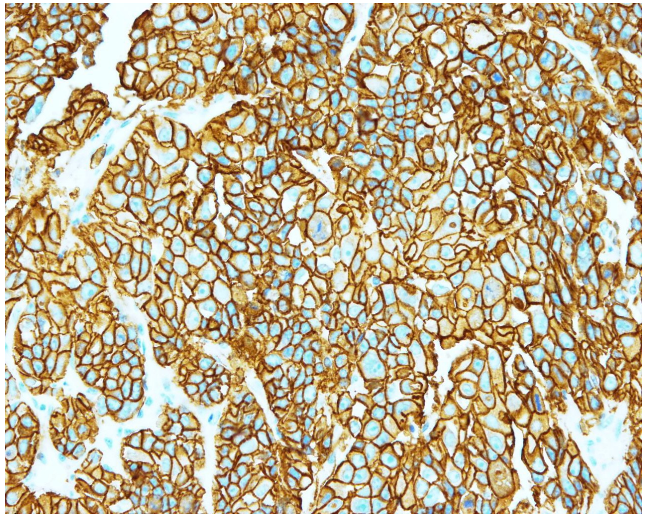

STO410 gastric cancer PDX model
HER2 IHC 3+

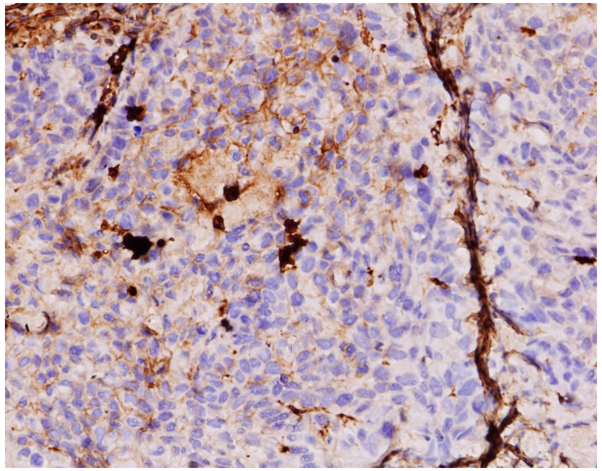

BC207 breast cancer PDX model
HER2 IHC 2+

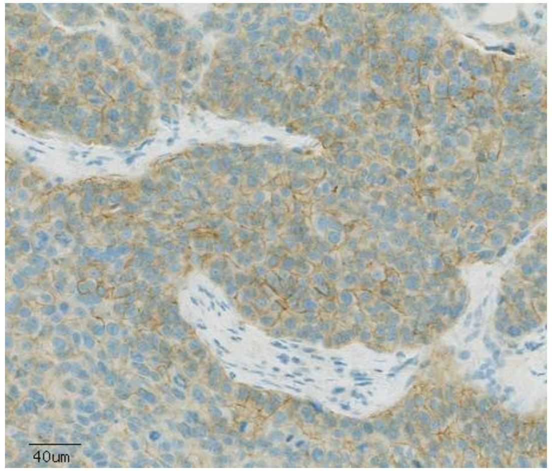

BC197 breast cancer PDX model
HER2 IHC 2+

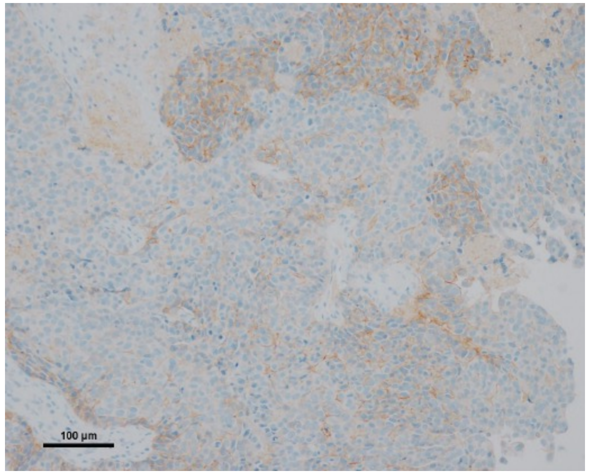

BC128 breast cancer PDX model
HER2 IHC 1+

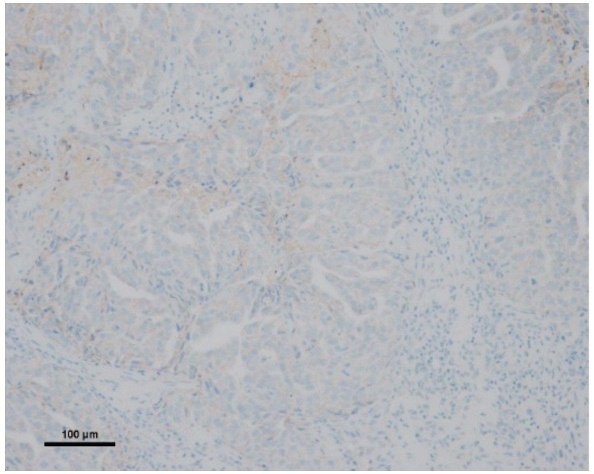

BC085 breast cancer PDX model
HER2 IHC 1+

Supplementary Fig. 13. HER2 immunohistochemistry (IHC) using antibody D8F12 (see Methods). HER2 status was determined by the Genentech Pathology Core lab using scores of 0, 1+, 2+ or 3+ as per 2013 ASCO CAP guidelines (2). IHC was performed once for each model.

Supplementary Fig. 13

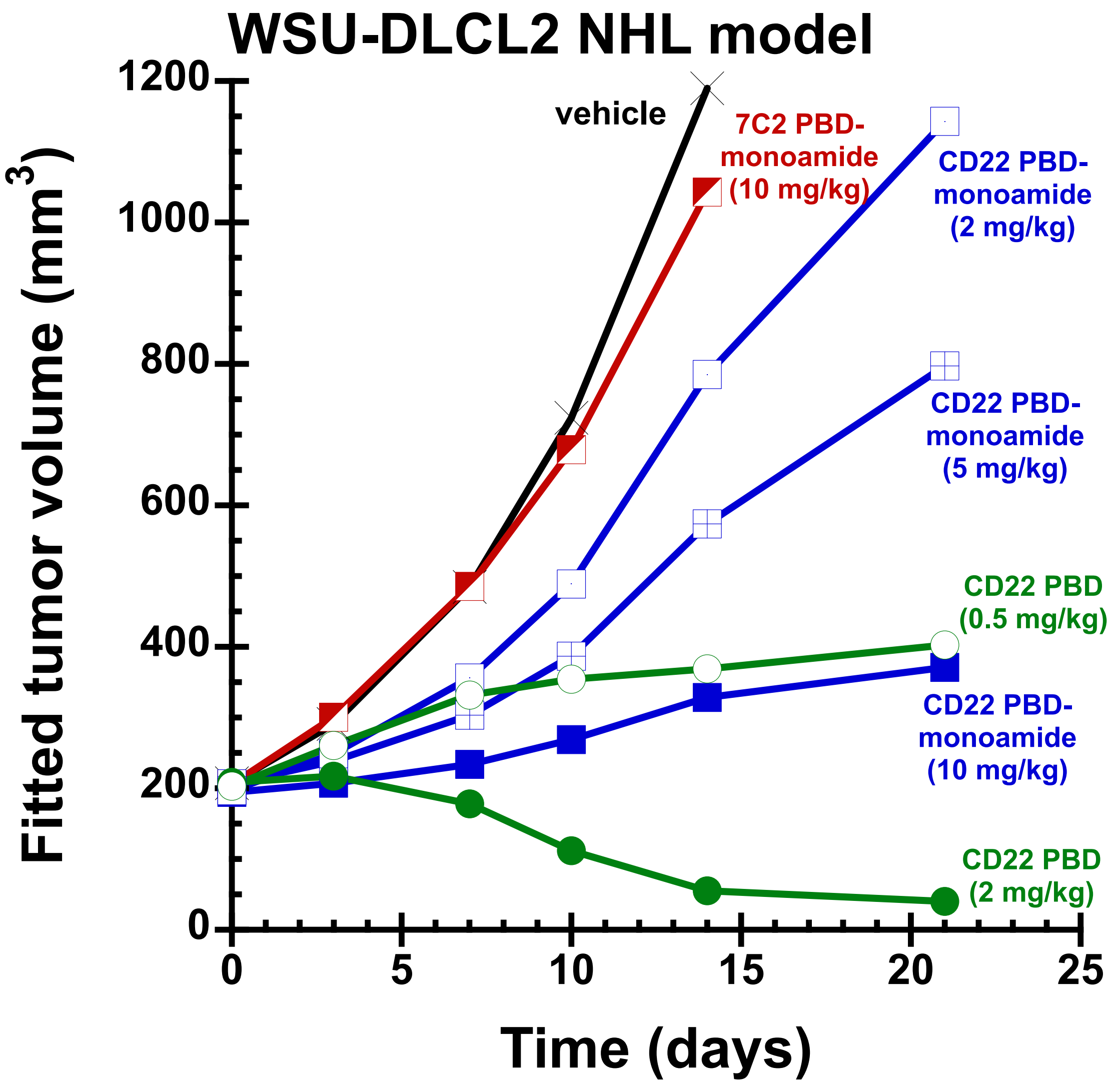

Supplementary Fig. 14. Efficacy study in WSU-DLCL2 Non-Hodgkin Lymphoma (NHL) model.
Mice were treated with CD22 PBD-monoamide (2, 5, 10 mg/kg single injection; n=6, n=7 and n=7 respectively); CD22 PBD (0.2, 5 mg/kg single injection; n=7 mice per group), anti-HER2 7C2 PBD-monoamide (10 mg/kg single injection; n=5 mice), or ADC vehicle (n=5 mice). Both CD22 conjugates demonstrated dose-dependent tumor growth inhibition, with no observed efficacy of the HER2 ADC. As WSU-DLCL2 cells do not express HER2, the results demonstrate lack of target-independent activity of 7C2 PBD-monoamide. Source data are provided as a Source Data file.

Supplementary Fig. 14

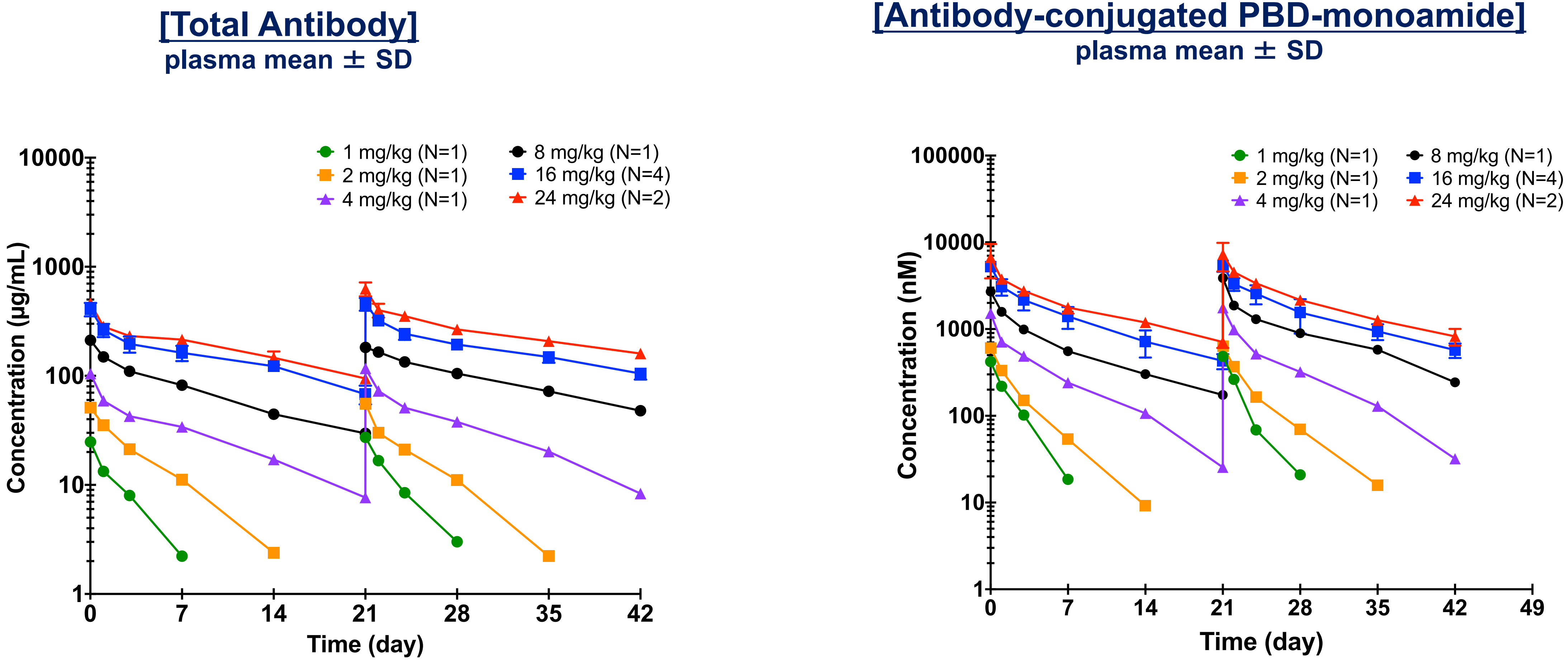

Supplementary Fig. 15. Pharmacokinetics of DHES0815A in cynomolgus monkeys.
DHES0815A was dosed at 1, 2, 4, 8, 16 and 24 mg/kg every 3 weeks for 2 total doses. Serum concentrations of total antibody (left panel) and antibody-conjugated PBD-monoamide (right panel) were assessed. Exposure was dose-proportional, with linearity at doses above 4 mg/kg. For 1, 2, 4, and 8 mg/kg doses, there was n=1 animal per group; n=4 for 16 mg/kg and n=2 for 24 mg/kg. Source data are provided as a Source Data file.

Supplementary Fig. 15

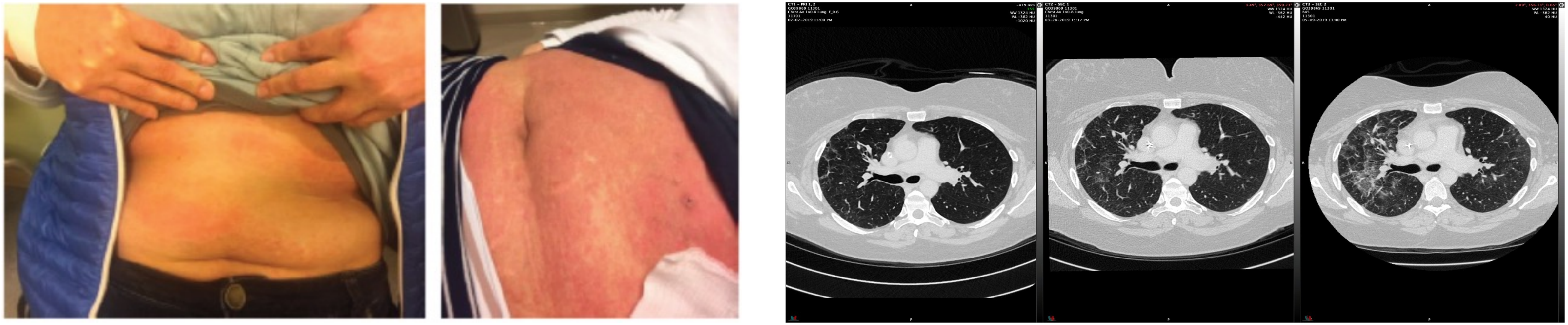

C3D8 (cycle 3/day 8)

C3D35 (cycle 3/day 35)

Screening

C2D15 (cycle 2/day 15) subtle GGO (ground glass opacity), right

C3D35 (cycle 3/day 35) bilateral GGO (ground glass opacity)

Supplementary Fig. 16. Example of pulmonary and dermal toxicities (in the same patient) .
A patient dosed at 4.0 mg/kg for 3 cycles presented with Grade 1 rash (left panels) and Grade 1 eyelid edema 4 days following Cycle 3 dosing. Both AEs worsened to Grade 2 10 days later. Dosing was held and a skin biopsy performed which revealed superficial vascular ectasia and proliferation, mild papillary edema, and focal sparse superficial perivascular lymphocytic infiltrate. While on dose hold, the patient presented with Grade 2 pneumonitis (35 days after the Cycle 3 dose; right panels), and was withdrawn from study treatment. The Grade 2 rash, eyelid edema and pneumonitis were ongoing at the end of study participation.

Supplementary Table 1. Cynomolgus monkey safety data for DHES0815A

Dose mg/kg	Clinical Observations	Clinical Pathology	Anatomic Pathology
**4**	darkened skin day 10 to end of study	↓ (mild) lymphocytes, eosinophils<br>• 1 week post dose, females only	Skin: hyperpigmentation (minimal)
**8**	as above, plus:<br>• slight corneal pigmentation (1/10 animals; day 88)	↓ (mild) lymphocytes, eosinophils<br>• 1 week post dose, females only	Skin: hyperpigmentation (minimal)
**12**	as above, plus:<br>• transient red skin (2/10 animals; day 43)<br>• slight corneal pigmentation (4/10 animals; days 66/68)	↓ (mild) lymphocytes, eosinophils<br>• 1 week post dose, females only, no clear recovery<br>↓ (mild) reticulocytes<br>• day 29 females only<br>• days 50, 71, 92 males and females; partial recovery	Skin: hyperpigmentation (minimal-mild); not reversible<br><br>Lymphoid (spleen, lymph node): lymphoid depletion (minimal); reversible<br><br>Lung: increased alveolar macrophages/alveolar degeneration, focal (minimal, n=1); not present in recovery animals

Supplementary Table 2. Patient demographics and disease characteristics

	All Patients (N=14)
Age, years, median (range)	55 (39-71)
Sex, female	14 (100%)
Race	
American Indian or Alaska Native	1 (7%)
Asian	3 (21%)
Black or African American	1 (7%)
White	9 (64%)
Baseline ECOG status	
0	6 (43%)
1	8 (57%)
HER2 Expression (central testing)*	
IHC 2+	5 (36%)
IHC 3+	8 (57%)
Not determined	1 (7%)
Hormone receptor status (local testing)	
Positive	8 (57%)
Negative	6 (43%)
Sites of metastatic disease	
Lymph node only	2 (14%)
Visceral (lung, liver)	9 (64%)
Other†	3 (21%)
Prior anti-HER2 regimens	
Trastuzumab	14 (100%)
Pertuzumab	10 (71%)
Trastuzumab emtansine	13 (93%)
Lapatinib	7 (50%)

Abbreviations: ECOG, Eastern Cooperative Oncology Group; IHC, immunohistochemistry.

* HER2 status meeting eligibility criteria were confirmed per local pathology results prior to study enrollment for all patients.

† One patient with lymph node and breast lesions, 1 patient with breast lesions, 1 patient with lymph node, pleural effusion, and bone lesions.

Supplementary Table 3. Summary of PK parameters total Ab, conjugated PBD-ma, unconjugated PBD-ma

**PK Parameters** *(Cycle 1)*	**HER2-ds-PBDMA 6 mg/kg (N=2)**	**HER2-ds-PBDMA 4 mg/kg (N=3)**	**HER2-ds-PBDMA 2.4 mg/kg (N=3)**	**HER2-ds-PBDMA 1.2 mg/kg (N=3)**	**HER2-ds-PBDMA 0.6 mg/kg (N=3)**
	Total Antibody [Mean (%CV)]	Total Antibody [Mean (%CV)]	Total Antibody [Mean (%CV)]	Total Antibody [Mean (%CV)]	Total Antibody [Mean (%CV)]
**Cmax** (μg/mL)	119 (0)	89.2 (11)	50.8 (23)	50.0 (28)	13.4 (28)
**$AUC_{0\text{-}inf}$** (day*μg/mL)	1278.6 (26)	862.3 (16)	241.5 (44)	162.4 (29)	42.7 (55)
**Clearance** (mL/day/kg)	6.2 (18)	6.0 (4.2)	12.0 (57)	8.0 (28)	16.6 (42)
**t1/2** (day)	9.2 (12)	9.5 (33)	2.5 (51)	3.6 (28)	1.28 (18)
**Vss** (L)	81 (6)	81.4 (30)	40.6 (49)	42.1 (49)	30.2 (43)
	acPBDMA [Mean (%CV)]	acPBDMA [Mean (%CV)]	acPBDMA [Mean (%CV)]	acPBDMA [Mean (%CV)]	acPBDMA [Mean (%CV)]
**Cmax** (μg/mL)	0.86 (4.4)	0.71 (3.9)	0.39 (26)	0.25 (10)	0.092 (24)
**$AUC_{0\text{-}inf}$** (day*μg/mL)	7.61 (21)	4.97 (12)	1.48 (41)	1.25 (17)	0.26 (52)
**Clearance** (mL/day/kg)	6.9 (14.6)	6.7 (4.3)	13.9 (53)	7.1 (19)	19.4 (39)
**t1/2** (day)	7.5 (17)	7.2 (34)	2.58(28)	3.36(19)	1.65 (12)
**Vss** (L)	73.8 (2.1)	69.5 (30)	47.5 (24)	34.6 (4)	44.8 (33)
	Unconjugated PBDMA [Mean (%CV)]	Unconjugated PBDMA [Mean (%CV)]	Unconjugated PBDMA [Mean (%CV)]	Unconjugated PBDMA [Mean (%CV)]	Unconjugated PBDMA [Mean (%CV)]
**Cmax** (ng/mL)	1.17 (19)	0.77 (9.1)	0.53 (38)	0.38 (42)	0.108 (15)
**$AUC_{0\text{-}inf}$** (day*ng/mL)	4.98 (6.1)	4.42 (29)	2.46 (45)	1.76 (3)	0.69 (14)
**t1/2** (day)	7.9 (17)	7.5 (8.9)	7.7 (71)	6.74 (15)	7.04 (49)

HER2-ds-PBDMA = anti-HER2-disulfide PBD-monoamide (DHES0815A); acPBDMA = antibody-conjugated PBD-monoamide; PBDMA = PBD-monoamide
Source data are provided as a Source Data file.

FACS gating strategies

- fig 2c (bystander experiment)
- Supp. figure 1 (DHFR-G8 cells)
- Supp. figure 2 (SK-BR-3 cells cross-blocking study)
- Supp. figure 12 (cell cycle analysis)

Gating for figure 2c
(bystander study)

MCF7red

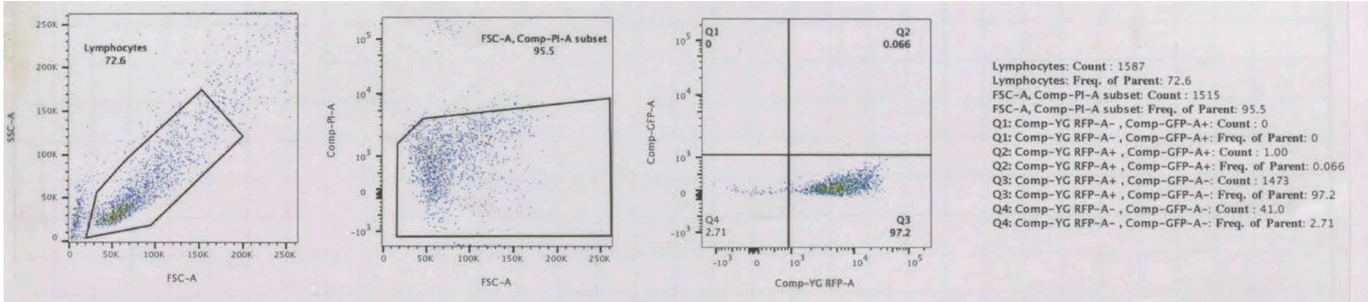

SK-BR-3/green

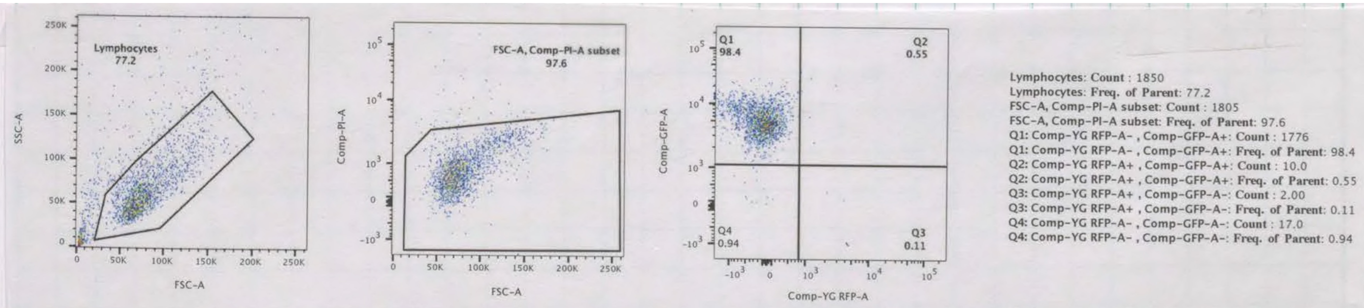

Mixed MCF7
and SK-BR-3

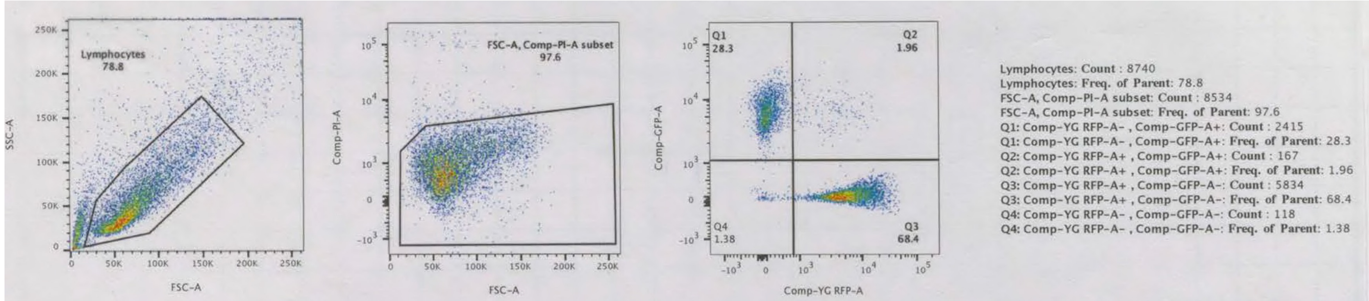

Sort on live cells
(PI-neg)

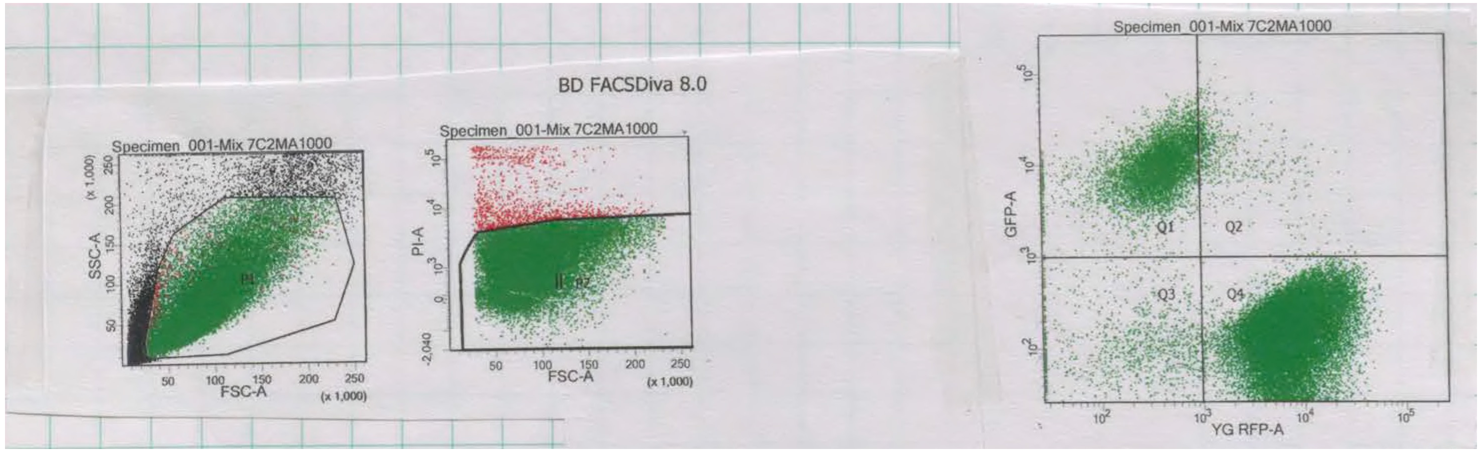

FACS gating for Supp. fig 1

DHFR-G8, murine isotype control

BD FACSDiva 8.0.1

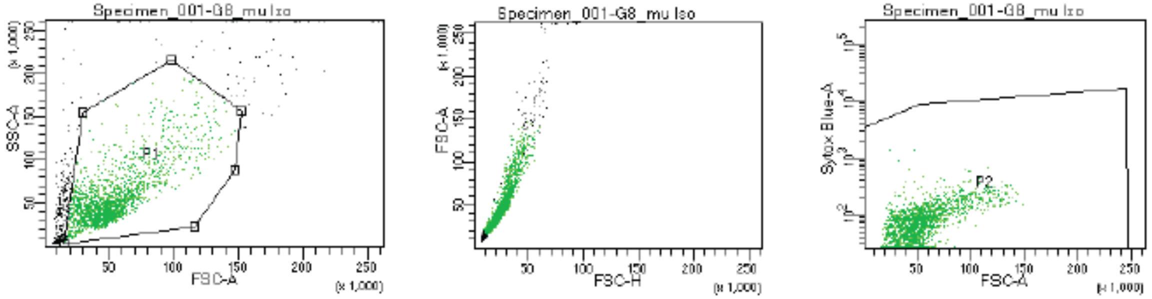

Tube: G8_mu Iso	#Events	%Parent	%Total
Population			
All Events	2,566	####	100.0
P1	2,138	83.3	83.3
P2	2,138	100.0	83.3
P4	14	0.7	0.5
P3	1,218	57.0	47.5

Experiment Name: JGuo_073119_DHFR G8_gdl Paper
Specimen Name: Specimen_001
Tube Name: G8_mu Iso
Record Date: Jul 31, 2019 7:49:14 PM
$OP: Administrator

Population	#Events	%Parent	%Total	PE-A Geo Mean	PE-A Mean	PE-A Median
All Events	2,566	####	100.0	####	9	3
P1	2,138	83.3	83.3	####	9	3
P2	2,138	100.0	83.3	####	9	3
P4	14	0.7	0.5	497	619	482
P3	1,218	57.0	47.5	10	21	11

DHFR-G8, human isotype control

BD FACSDiva 8.0.1

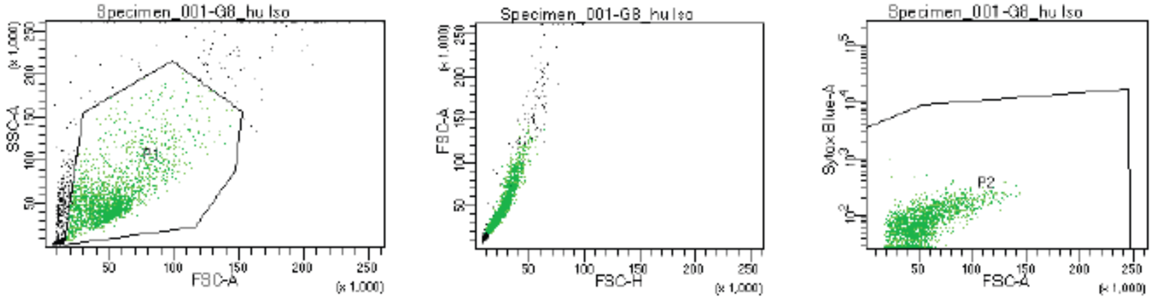

Tube: G8_hu Iso	#Events	%Parent	%Total
Population			
All Events	2,709	####	100.0
P1	2,174	80.3	80.3
P2	2,174	100.0	80.3
P4	1	0.0	0.0
P3	1,026	47.2	37.9

Experiment Name: JGuo_073119_DHFR G8_gdl Paper
Specimen Name: Specimen_001
Tube Name: G8_hu Iso
Record Date: Jul 31, 2019 7:51:50 PM
$OP: Administrator

Population	#Events	%Parent	%Total	PE-A Geo Mean	PE-A Mean	PE-A Median
All Events	2,709	####	100.0	####	3	1
P1	2,174	80.3	80.3	####	2	0
P2	2,174	100.0	80.3	####	2	0
P4	1	0.0	0.0	718	718	718
P3	1,026	47.2	37.9	9	13	9

FACS gating for Supp. fig 2

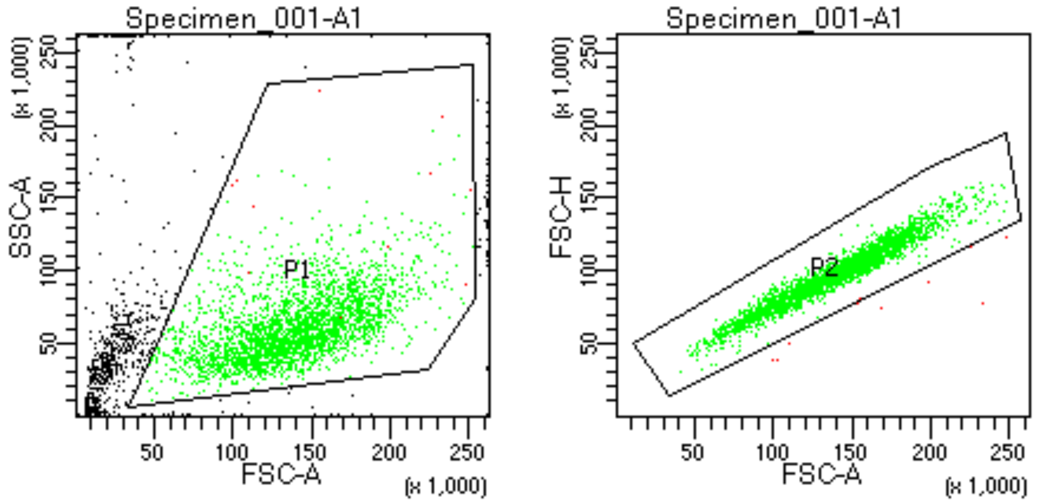

Well: A1

Population	#Events	%Parent	%Total
All Events	3,837	####	100.0
P1	2,877	75.0	75.0
P2	2,865	99.6	74.7
Positive	2,840	99.1	74.0

Experiment Name:	JunGuo_042319_S3_Cross Blo...
Specimen Name:	Specimen_001
Well Name:	A1
Record Date:	Apr 23, 2019 8:24:29 PM
$OP:	Administrator

Population	#Events	%Parent	YG PE-A Geo Mean	YG PE-A Mean	YG PE-A Median
P2	2,865	99.6	####	90	84
Positive	2,840	99.1	77	91	85

Gating for Supp. Fig. 12

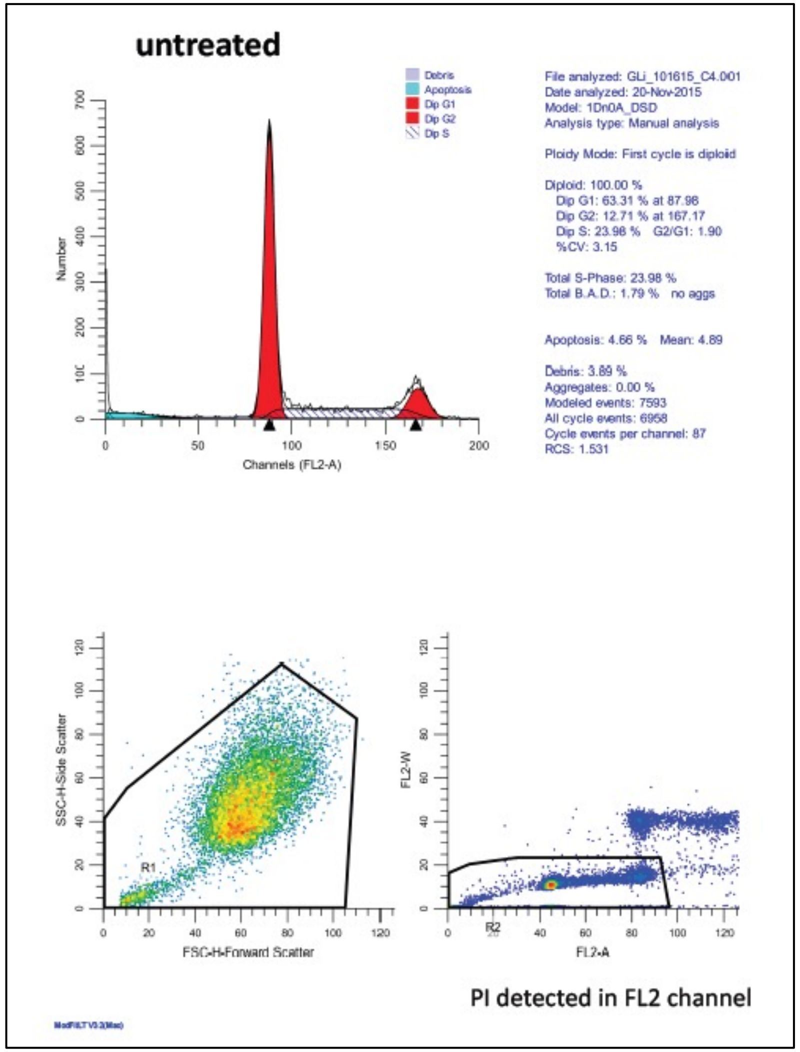

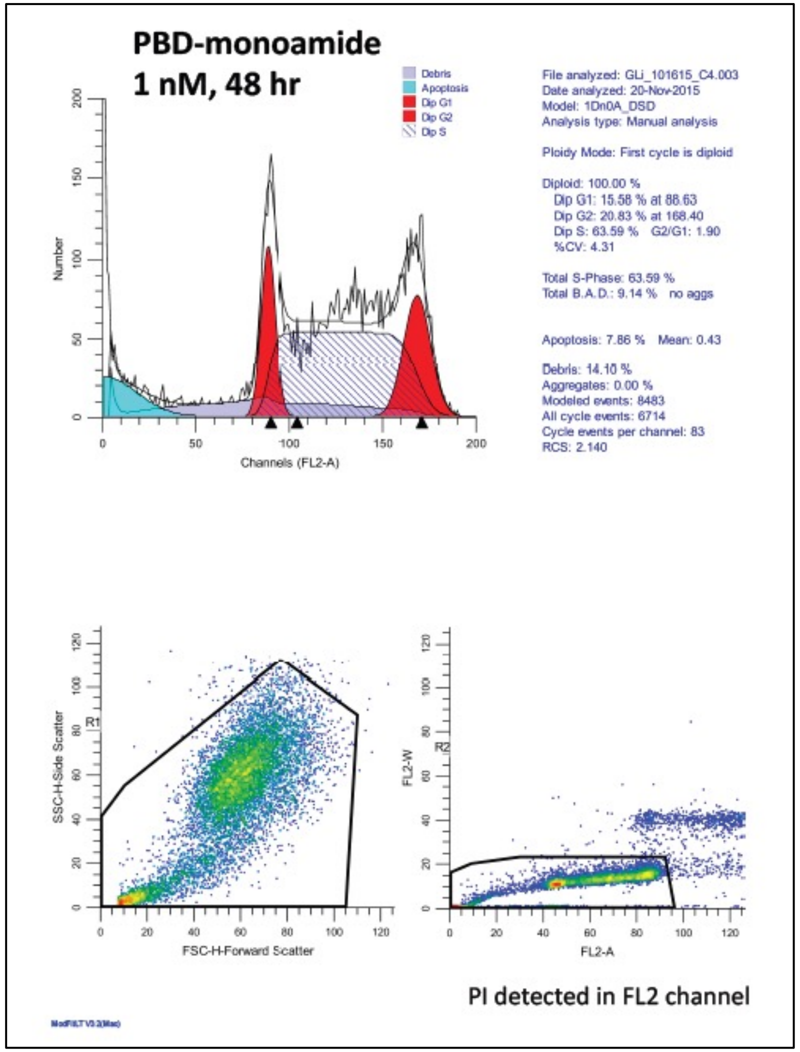

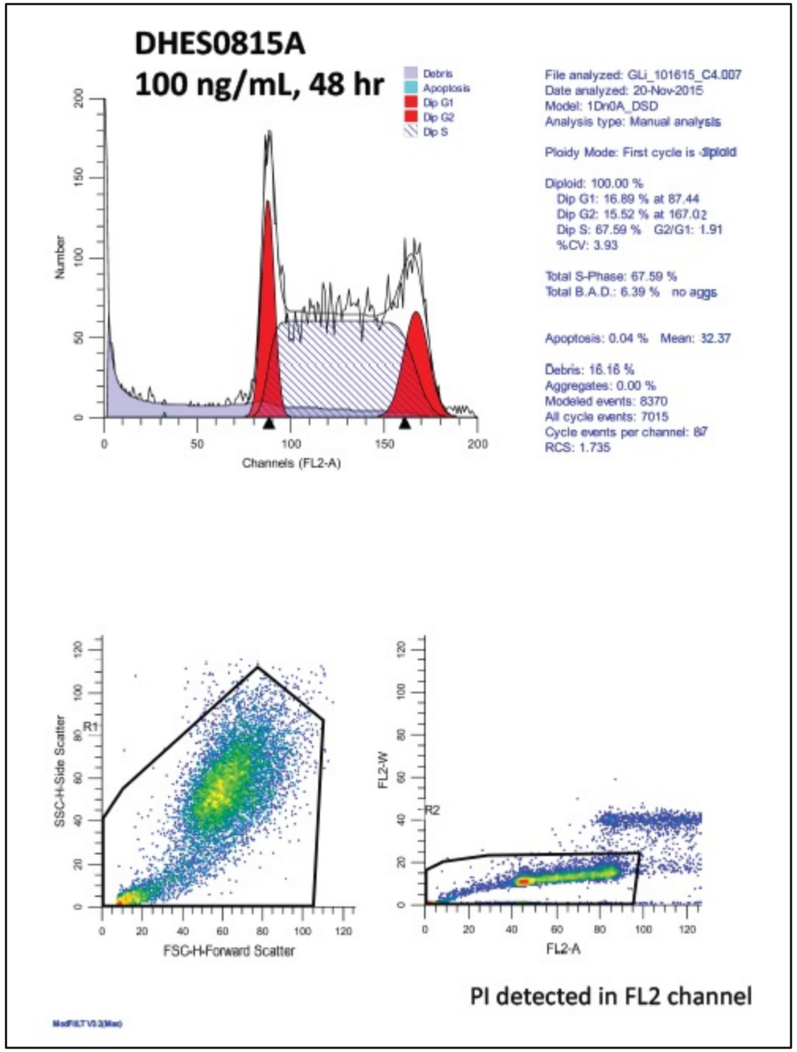

Supplementary references

1. Lewis Phillips, et al. Trastuzumab does not bind rat or mouse ErbB2/neu: implications for selection of non-clinical safety models for trastuzumab-based therapeutics. *Breast Cancer Res. Treat.* **191**, 303-317 (2022).

2. Wolff, A.C., et al. Recommendations for Human Epidermal Growth Factor Receptor 2 testing in breast cancer: American Society of Clinical Oncology/College of American Pathologists clinical practice guideline update. *J. Clin. Oncology* **31**, 3997-4014 (2013).
