# Supplementary material for: The HER2-directed antibody-drug conjugate DHES0815A in advanced and/or metastatic breast cancer: preclinical characterization and phase 1 trial results
Source: Nat Commun. 2024 Jan 11;15:466. doi: 10.1038/s41467-023-44533-z (PMC10784567; doi:10.1038/s41467-023-44533-z)
Supplement: Supplementary file 2 — Reporting Summary [file 41467_2023_44533_MOESM2_ESM.pdf]

nature portfolio

Corresponding author(s): *Double-anonymous peer review submissions: write DAPR and your manuscript number here instead of author names.*

Last updated by author(s): *YYYY-MM-DD*

Reporting Summary

Nature Portfolio wishes to improve the reproducibility of the work that we publish. This form provides structure for consistency and transparency in reporting. For further information on Nature Portfolio policies, see our Editorial Policies and the Editorial Policy Checklist.

Statistics

For all statistical analyses, confirm that the following items are present in the figure legend, table legend, main text, or Methods section.

n/a	Confirmed	
☐	☒	The exact sample size (*n*) for each experimental group/condition, given as a discrete number and unit of measurement
☐	☒	A statement on whether measurements were taken from distinct samples or whether the same sample was measured repeatedly
☒	☐	The statistical test(s) used AND whether they are one- or two-sided<br>*Only common tests should be described solely by name; describe more complex techniques in the Methods section.*
☒	☐	A description of all covariates tested
☒	☐	A description of any assumptions or corrections, such as tests of normality and adjustment for multiple comparisons
☐	☒	A full description of the statistical parameters including central tendency (e.g. means) or other basic estimates (e.g. regression coefficient) AND variation (e.g. standard deviation) or associated estimates of uncertainty (e.g. confidence intervals)
☒	☐	For null hypothesis testing, the test statistic (e.g. *F*, *t*, *r*) with confidence intervals, effect sizes, degrees of freedom and *P* value noted<br>*Give P values as exact values whenever suitable.*
☒	☐	For Bayesian analysis, information on the choice of priors and Markov chain Monte Carlo settings
☒	☐	For hierarchical and complex designs, identification of the appropriate level for tests and full reporting of outcomes
☒	☐	Estimates of effect sizes (e.g. Cohen's *d*, Pearson's *r*), indicating how they were calculated

Our web collection on statistics for biologists contains articles on many of the points above.

Software and code

Policy information about availability of computer code

Data collection	For laboratory studies: Envision Multilabel Platereader, Guava EasyCyte Cytometer, IncuCyte Live-Cell Analysis System, Fortessa Flow Cytometer, Packard Gamma Counter, Cool SNAP HQ2 CCd Camera, LC/MS Sciet Triple TCF, FACSCalibur, FACSymphony A5. For the phase 1 study, Medidata Rave software.
Data analysis	Kaleidagraph software, IncuCyte Live-Cell Analysis System, Fortessa Flow Cytometer, Guava EasyCyte, New Ligand (for Scatchard). For the phase 1 study, SAS v9.4 was used to generate Clinical Report outputs by SPA.

For manuscripts utilizing custom algorithms or software that are central to the research but not yet described in published literature, software must be made available to editors and reviewers. We strongly encourage code deposition in a community repository (e.g. GitHub). See the Nature Portfolio guidelines for submitting code & software for further information.

Data

Policy information about availability of data

All manuscripts must include a data availability statement. This statement should provide the following information, where applicable:

- Accession codes, unique identifiers, or web links for publicly available datasets
- A description of any restrictions on data availability
- For clinical datasets or third party data, please ensure that the statement adheres to our policy

All data generated or analysed for the studies in this manuscript are included in the paper and the supplementary files. Qualified researchers may request access to individual patient level data through the clinical study data request platform (https://vivli.org/). Further details on Roche's criteria for eligible studies are available

here: https://vivli.org/members/ourmembers/. For further details on Roche's Global Policy on the Sharing of Clinical Information and how to request access to related clinical study documents, see https://www.roche.com/innovation/process/clinical-trials/data-sharing/. Source data are provided with this paper.
Qualified researchers may request access to individual patient level data through the clinical study data request platform (https://vivli.org/). Further details on Roche's criteria for eligible studies are available here: https://vivli.org/members/ourmembers/. For further details on Roche's Global Policy on the Sharing of Clinical Information and how to request access to related clinical study documents, see https://www.roche.com/innovation/process/clinical-trials/data-sharing/. Source data are provided with this paper.

Research involving human participants, their data, or biological material

Policy information about studies with human participants or human data. See also policy information about sex, gender (identity/presentation), and sexual orientation and race, ethnicity and racism.

Reporting on sex and gender	We did not specifically collect data on sex or gender. All 14 patients enrolled were documented as female (eligible patients were not selected based on sex/gender).
Reporting on race, ethnicity, or other socially relevant groupings	The manuscript reports on race, as reported by the site and no other socially relevant groupings. The groupings were not used for any analyses as the study only enrolled 14 patients.
Population characteristics	The study enrolled 14 patients (median age 55). All 14 patients were documented as female. The baseline ECOG status was either 0 (43%) or 1 (57%). HER2 expression in tumor was assessed in 13 of 14 patients and found to be either 2+ (36%) or 3+ (57%). Hormone receptor status was positive in 57% and negative in 43%. 14% had metastatic disease only to lymph nodes and 64% had metastases in visceral organs. All patients received prior trastuzumab therapy, 71% received prior pertuzumab, 93% received prior traztuzumab emtansine and 50% had prior lapatinib.
Recruitment	Study sites used their existing patient population to determine which subjects met inclusion/exclusion criteria and then invited them to review the informed consent form. The study was open for recruitment at 6 urban, academic centers in the US and South Korea, thus the patients enrolled were those who could access these centers. This may have resulted in selection bias for patients who have the greatest access to advanced medical care; for example, several had participated in other clinical trials prior to coming onto this study. Patients were also required to have progressed on prior HER2 therapy and still have a good performance status (ECOG 0 or 1) to participate on this study; this may have led to a survivorship bias.
Ethics oversight	The study was conducted in accordance with the Declaration of Helsinki and the principles of Good Clinical Practice. The protocol was approved by institutional review boards and ethic committees from each study site (Dana Farber Cancer Institute, Memorial Sloan Kettering Cancer Center, Yale Cancer Center, Asan Medical Center, Columbia Medical Center, and Sarah Cannon Research Institute), and all patients provided written informed consent before undergoing any study procedures.

Note that full information on the approval of the study protocol must also be provided in the manuscript.

Field-specific reporting

Please select the one below that is the best fit for your research. If you are not sure, read the appropriate sections before making your selection.

- [x] Life sciences
- [ ] Behavioural & social sciences
- [ ] Ecological, evolutionary & environmental sciences

For a reference copy of the document with all sections, see nature.com/documents/nr-reporting-summary-flat.pdf

Life sciences study design

All studies must disclose on these points even when the disclosure is negative.

Sample size	The phase 1 study used a standard 3+3 design in dose escalation. The exact sample size for this trial was determined by the number and size of the cohorts needed per the dose-escalation and cohort expansion rules. Sample sizes for in vitro studies were n=3 or n=4 replicates per treatment group to generate means and standard deviations/errors. Sample size in mouse tumor studies were n=5, 6, 7, 8, or 10 per treatment group to take into account inter-group variability.
Data exclusions	No data were excluded from any studies in this manuscript.
Replication	For the phase 1 study, only patients with HER2-positive breast cancer were included and HER2 status was centrally confirmed. All in vitro studies were repeated 3-6 times. with similar results. In vivo mouse tumor studies using cell line or the Fo5 model were performed 2-3 times with similar results. PDX models were performed once (done at a CRO).
Randomization	For the phase 1 study, participants were not randomized due to the early stage (phase 1) of the study. For in vivo mouse tumor studies, mice were randomly divided into groups after tumor reached a certain size (~150-300 mm3).
Blinding	Blinding was not relevant due to the early stage (phase 1) of the trial. This was a single-arm trial with no control arm. No blinding was used for cell culture or animal studies.

Reporting for specific materials, systems and methods

We require information from authors about some types of materials, experimental systems and methods used in many studies. Here, indicate whether each material, system or method listed is relevant to your study. If you are not sure if a list item applies to your research, read the appropriate section before selecting a response.

Materials & experimental systems

n/a	Involved in the study
☐	☒ Antibodies
☐	☒ Eukaryotic cell lines
☒	☐ Palaeontology and archaeology
☐	☒ Animals and other organisms
☐	☒ Clinical data
☒	☐ Dual use research of concern
☒	☐ Plants

Methods

n/a	Involved in the study
☒	☐ ChIP-seq
☐	☒ Flow cytometry
☒	☐ MRI-based neuroimaging

Antibodies

Antibodies used	Antibodies (1:1000 dilution) included phospho-H2AX (Ser139) (cat#2577), phospho-p53 (Ser15) (cat# 9286), p53 (cat# 2527), phospho-Histone H3 (Ser10) (cat# 9701), PARP (cat# 9541) and b-actin (cat# 5125), all obtained from Cell Signal Technology. FACS antibodies were from Jackson ImmunoResearch: Goat Anti-Mu-PE, Cat #: 115-116-146 and Goat Anti-Hu-PE, Cat #: 109-116-088, both diluted 1 to 1,000 . For immunofluorescence studies: 0.5 ug/mL anti-LAMP1 (Becton Dickinson #555798), 1.9 ug/mL anti-Cy3 human IgG (Jackson ImmunoResearch #709-166-149), 1.9 ug/mL Alexa Fluor 647 anti-mouse (Jackson ImmunoResearch #715-606-150). Trastuzumab and 7C2 variants were made at Genentech.
Validation	All antibodies were validated by the vendor or at Genentech for binding, species selectivity and application. All antibodies were further tested to determine optimal concentrations/dilutions for each application.

Eukaryotic cell lines

Policy information about cell lines and Sex and Gender in Research

Cell line source(s)	All tumor cell lines were from The American Type Culture Collection (ATCC), except BJAB (German Collection of Microorganisms and Cell Cultures, DSMZ) and KPL-4 (gift from J. Kurebayashi, Okayama, Japan). All cells were deposited into an internal Genentech cell bank for maintenance and routine testing for mycoplasma and cell identity. Normal cells were obtained from LifeLine Cell Technologies. Cell lines used were: KPL-4, SK-BR-3, MCF7, MDA-MB-468, SW900, NCI-H1781, HCC1937, T-47D, HCC1569X2, BJAB, MES-SA, MES-SA/Dx5, BT-474, CHO-HER2, HCC1569, HCC1954.
Authentication	short tandem repeat (STR) profiling; single nucleotide polymorphism (SNP) fingerprinting
Mycoplasma contamination	Cells were routinely tested and found negative for mycoplamsa contamination
Commonly misidentified lines (See ICLAC register)	none of the cell lines included in this manuscript are listed on ICLAC.

Animals and other research organisms

Policy information about studies involving animals; ARRIVE guidelines recommended for reporting animal research, and Sex and Gender in Research

Laboratory animals	mouse, female: 11-16 weeks of age for C.B-17 SCID.bg, Nu/Nu, and NSG; 6 weeks of age for Balb/c nude mice.
Wild animals	no wild animals were used in our studies
Reporting on sex	Human breast cancer cells used were all of human female origin. There are no available male breast cancer cell lines. Mouse gender was female for all studies.
Field-collected samples	No field collected samples used for this manuscript.
Ethics oversight	All animal studies were approved by Genentech and the Institutional Animal Care and Use Committee (IACUC) and adhered to the NIH Guidelines for the Care and Use of Laboratory Animals

Note that full information on the approval of the study protocol must also be provided in the manuscript.

Clinical data

Policy information about clinical studies
All manuscripts should comply with the ICMJE guidelines for publication of clinical research and a completed CONSORT checklist must be included with all submissions.

Clinical trial registration	Registered on clinicaltrials.gov as NCT03451162
Study protocol	The results posting, including the redacted protocol, were submitted to ClinicalTrials.go on 23June2022. NIH would typically review results records within 30 days of submission, but they have yet to review this submission. Therefore, the results and redacted protocol are still not available on the clinicaltrials.gov public website.
Data collection	US and South Korea, April 2-18-April 2019. Study visits occurred between April 2018 and July 2021 and data were entered into electronic case report forms (eCRF). Specific sites: Columbia University Medical Center, New York, NY; Memorial Sloan Kettering Cancer Center, New York, NY; Asan Medical Center, University of Ulsan College of Medicine, Seoul, Korea; Sarah Cannon Research Institute/Tennessee Oncology, Nashville, TN; Yale Cancer Center, Yale University, New Haven, CT
Outcomes	The primary and secondary outcomes were predefined by the Sponsor in discussion with the study investigators. The safety outcomes were assessed based on safety events, laboratory assessments and vital signs as reported by the study investigators in the electronic database. Adverse events were characterized according to the Common Terminology Criteria for Adverse events (CTCAE) v4.0. The efficacy outcomes are based on investigator assessment of CT scans using the RECIST v1.1 criteria. The PK outcomes are assessed via the PK analytes reported by the central laboratory

Flow Cytometry

Plots

Confirm that:

- [x] The axis labels state the marker and fluorochrome used (e.g. CD4-FITC).
- [x] The axis scales are clearly visible. Include numbers along axes only for bottom left plot of group (a 'group' is an analysis of identical markers).
- [x] All plots are contour plots with outliers or pseudocolor plots.
- [x] A numerical value for number of cells or percentage (with statistics) is provided.

Methodology

Sample preparation	Binding assays: adherent cell lines were detached, washed and resuspended in sample buffer with appropriate primary and secondary antibodies. Propidium Iodide (PI) was added to exclude non-viable cells. For cell cycle analysis, cells were detached after treatment. Both adherent and floating cells were collected, washed, fixed in cold methanol and treated with PI/RNAse staining buffer for analysis of cell cycle phases.
Instrument	Becton Dickinson FACSymphony A5 and FACSCalibur
Software	FloJo, ModFIt LT
Cell population abundance	FACS studies in Supp. figures 1 and 2 were simply to determine antibody binding to cell surface receptors on 2 cell lines (DHFR-G8 and SK-BR-3, respectively). As such, the relevant cell populations are just the 2 different cell lines, whose identify was verified by STR. These studies did not involve sorting. For the cell cycle analysis (Supp. fig. 12), abundance of cells in each phase of the cell cycle was determined by DNA content (PI staining). No sorting was involved in this study.
Gating strategy	FSC/SSC gates were first used to remove cell debris and doublets as standard protocol. This population was then gated for live/dead cells based on PI exclusion such that only live cells were analyzed for cell surface binding of antibodies. The population was then analuzed for PE signal shift, aligning with the histogram peak. Boundaries between positive and negative cells were defined by location of the histogram peaks.

- [x] Tick this box to confirm that a figure exemplifying the gating strategy is provided in the Supplementary Information.
