# Supplementary material for: The HER2-directed antibody-drug conjugate DHES0815A in advanced and/or metastatic breast cancer: preclinical characterization and phase 1 trial results
Source: Nat Commun. 2024 Jan 11;15:466. doi: 10.1038/s41467-023-44533-z (PMC10784567; doi:10.1038/s41467-023-44533-z)
Supplement: Supplementary file 4 — Source Data [file 41467_2023_44533_MOESM4_ESM.zip › source data files/phase 1/Best_target_sld_change_from_baseline.pdf]

Initial assig	Highest adı	Subject ID	Instance Nı	Subject Dis	Study Day (	Target SLD	Target SLD % change fr
0.6 mg/kg	2.4 mg/kg	11001	Screening		-14	19	
			Cycle 4 Tur	Yes	80	16	-0.158
0.6 mg/kg	0.6 mg/kg	11002	Screening		-5	113	
			Subject Dis	Yes	66	88	-0.221
0.6 mg/kg	0.6 mg/kg	11003	Screening		-6	280	
			Cycle 4 Tur	Yes	69	274.9	-0.018
1.2 mg/kg	1.2 mg/kg	11101	Screening		-12	111.4	
			Cycle 2 Tur	Yes	36	116.4	0.045
1.2 mg/kg	2.4 mg/kg	11102	Screening		-13	42.7	
			Cycle 8 Tur	Yes	201	26.6	-0.377
1.2 mg/kg	1.2 mg/kg	11103	Screening		-6	35.9	
			Cycle 38 Tu	Yes	832	0	-1
2.4 mg/kg	2.4 mg/kg	11201	Screening		-5	86	
			Cycle 2 Tur	Yes	38	84	-0.023
2.4 mg/kg	2.4 mg/kg	11202	Screening		-26	79.91	
			Cycle 2 Tur	Yes	38	118	0.477
2.4 mg/kg	2.4 mg/kg	11203	Screening		-14	82	
			Cycle 2 Tur	Yes	42	109	0.329
4.0 mg/kg	4.0 mg/kg	11301	Screening		-14	58.9	
			Unschedule	Yes	78	61	0.036
4.0 mg/kg	4.0 mg/kg	11302	Screening		-14	116	
			Cycle 4 Tur	Yes	85	124	0.069
4.0 mg/kg	4.0 mg/kg	11303	Screening		-27	51	
			Subject Dis	Yes	135	19	-0.627
6.0 mg/kg	6.0 mg/kg	11401	Screening		-9	18.8	
			Cycle 2 Tur	Yes	36	17.3	-0.08
6.0 mg/kg	6.0 mg/kg	11402	Screening		-2	32.9	
			Cycle 2 Tur	Yes	42	22.9	-0.304
