# Supplementary material for: The HER2-directed antibody-drug conjugate DHES0815A in advanced and/or metastatic breast cancer: preclinical characterization and phase 1 trial results
Source: Nat Commun. 2024 Jan 11;15:466. doi: 10.1038/s41467-023-44533-z (PMC10784567; doi:10.1038/s41467-023-44533-z)
Supplement: Supplementary file 4 — Source Data [file 41467_2023_44533_MOESM4_ESM.zip › source data files/in vitro/Fig 1b DNA binding of PBD compounds for Gail.pptx]

## Slide 1
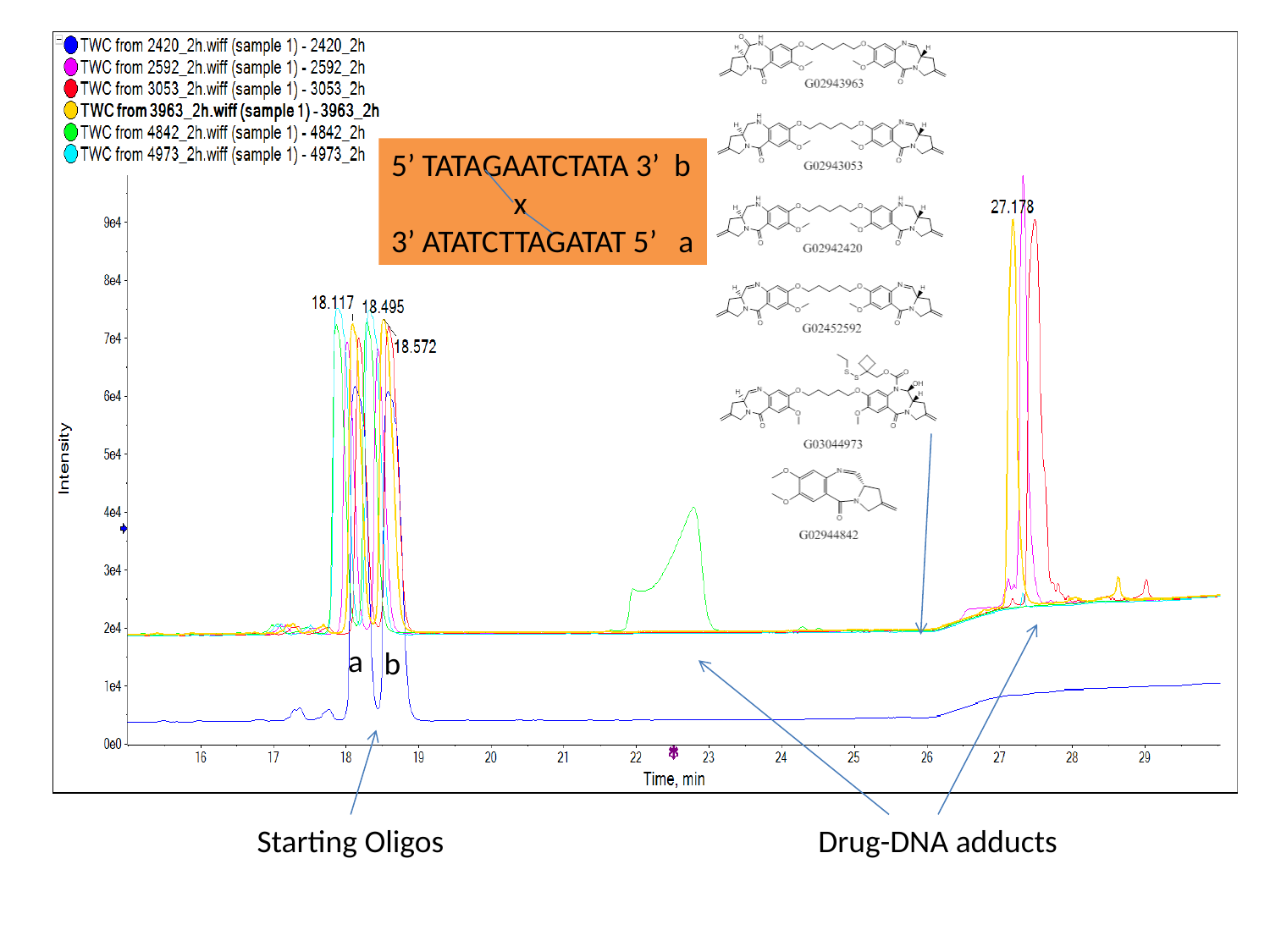

5’ TATAGAATCTATA 3’ b
 x
3’ ATATCTTAGATAT 5’ a
a
b
Starting Oligos
Drug-DNA adducts

## Slide 2
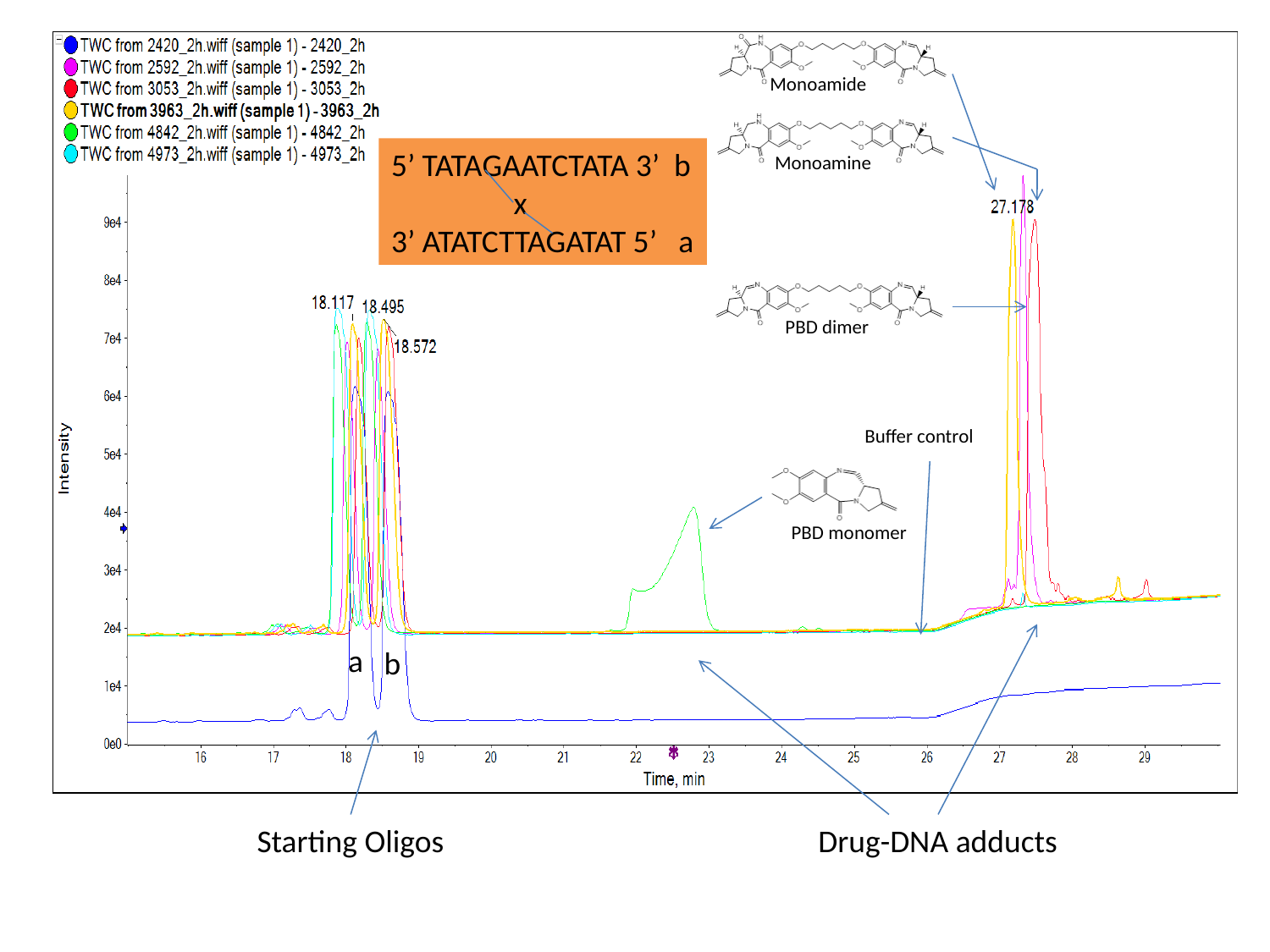

Monoamide
5’ TATAGAATCTATA 3’ b
 x
3’ ATATCTTAGATAT 5’ a
Monoamine
PBD dimer
Buffer control
PBD monomer
a
b
Starting Oligos
Drug-DNA adducts
