# Supplementary material for: The HER2-directed antibody-drug conjugate DHES0815A in advanced and/or metastatic breast cancer: preclinical characterization and phase 1 trial results
Source: Nat Commun. 2024 Jan 11;15:466. doi: 10.1038/s41467-023-44533-z (PMC10784567; doi:10.1038/s41467-023-44533-z)
Supplement: Supplementary file 4 — Source Data [file 41467_2023_44533_MOESM4_ESM.zip › source data files/in vitro/Fig3 western original images .pptx]

## Slide 1
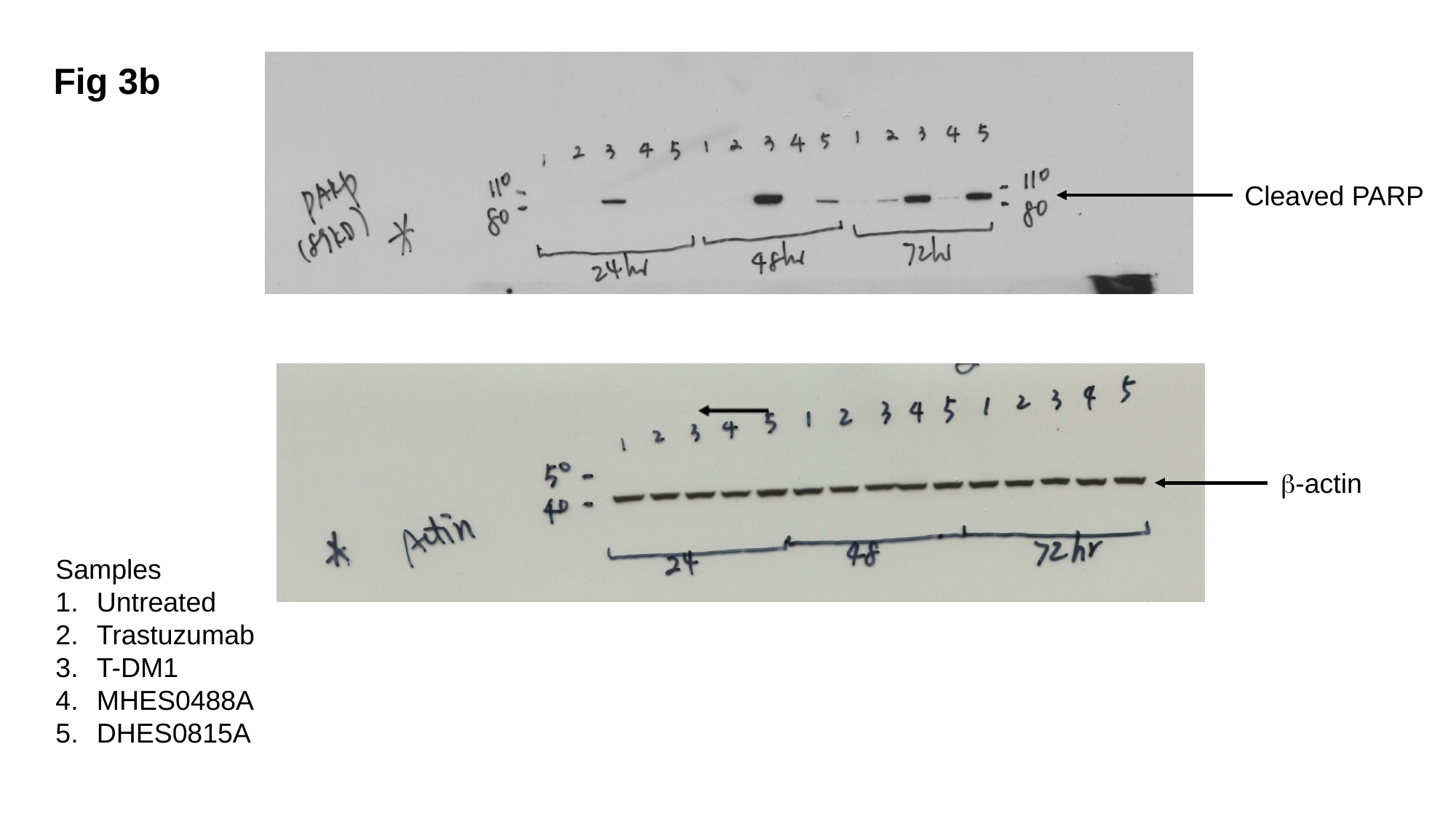

Fig 3b
Cleaved PARP
b-actin
Samples
Untreated
Trastuzumab
T-DM1
MHES0488A
DHES0815A

## Slide 2
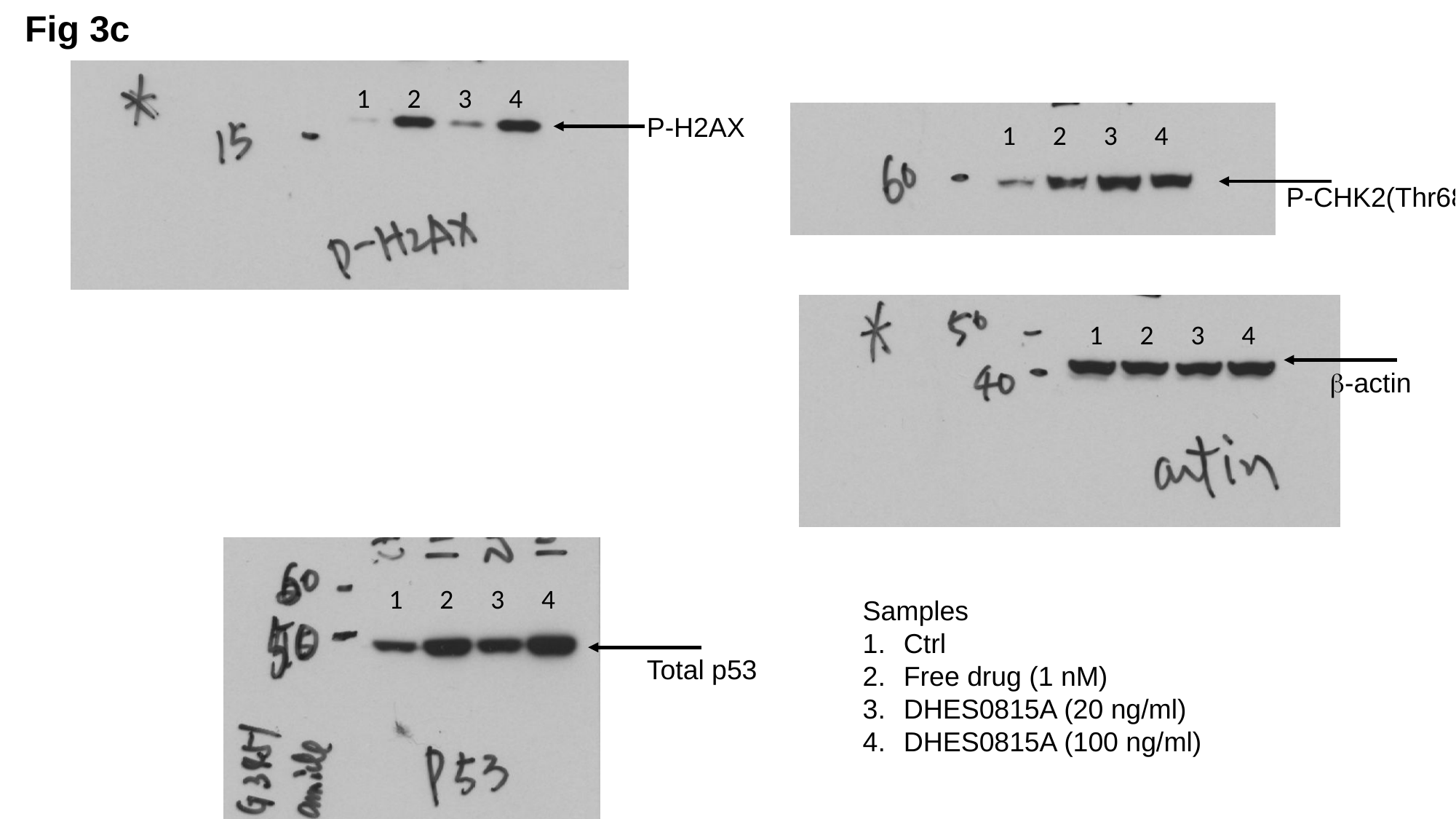

Fig 3c
1 2 3 4
P-H2AX
1 2 3 4
P-CHK2(Thr68)
1 2 3 4
b-actin
1 2 3 4
Samples
Ctrl
Free drug (1 nM)
DHES0815A (20 ng/ml)
DHES0815A (100 ng/ml)
Total p53

## Slide 3
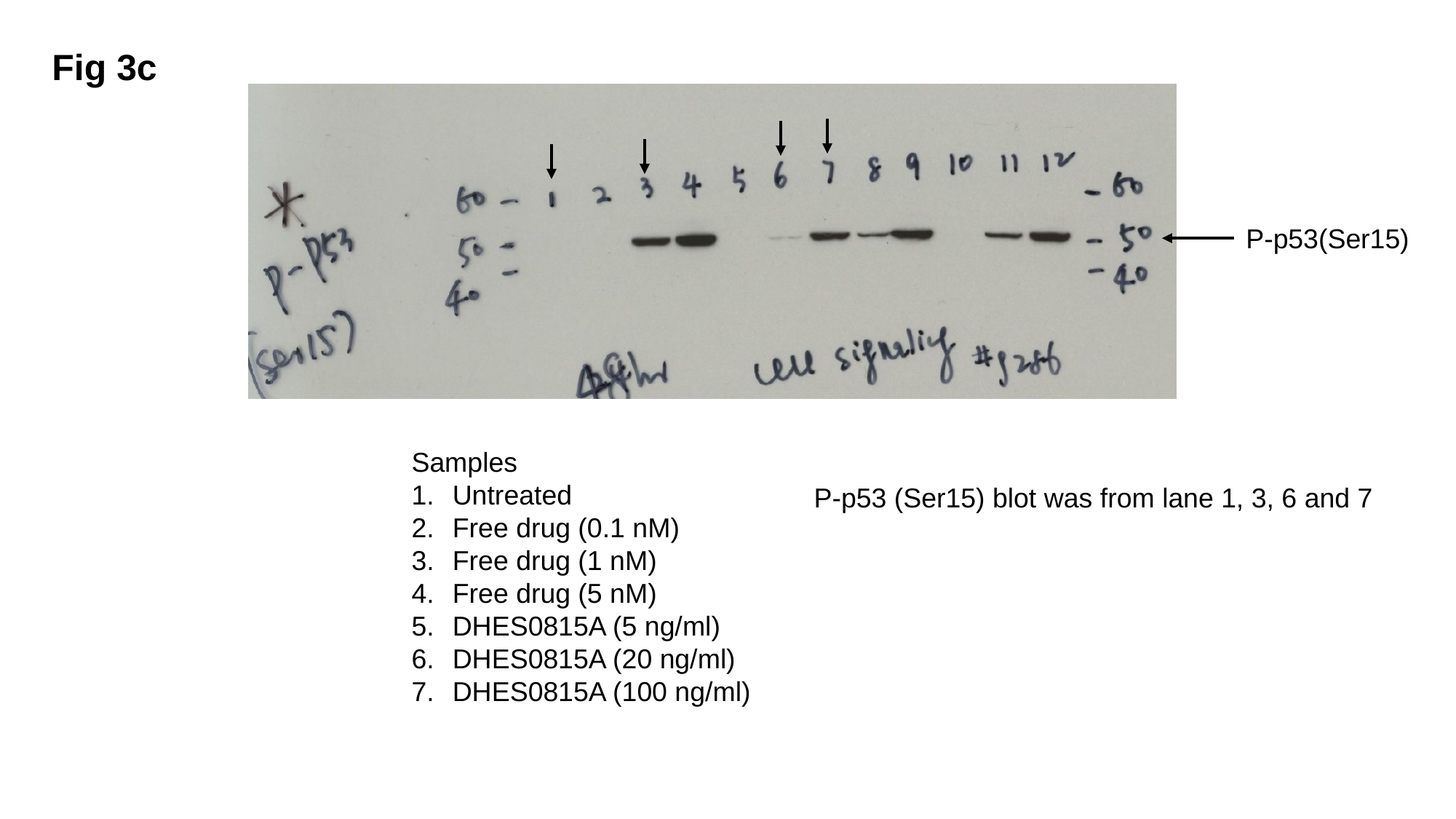

Fig 3c
P-p53(Ser15)
Samples
Untreated
Free drug (0.1 nM)
Free drug (1 nM)
Free drug (5 nM)
DHES0815A (5 ng/ml)
DHES0815A (20 ng/ml)
DHES0815A (100 ng/ml)
P-p53 (Ser15) blot was from lane 1, 3, 6 and 7

## Slide 4
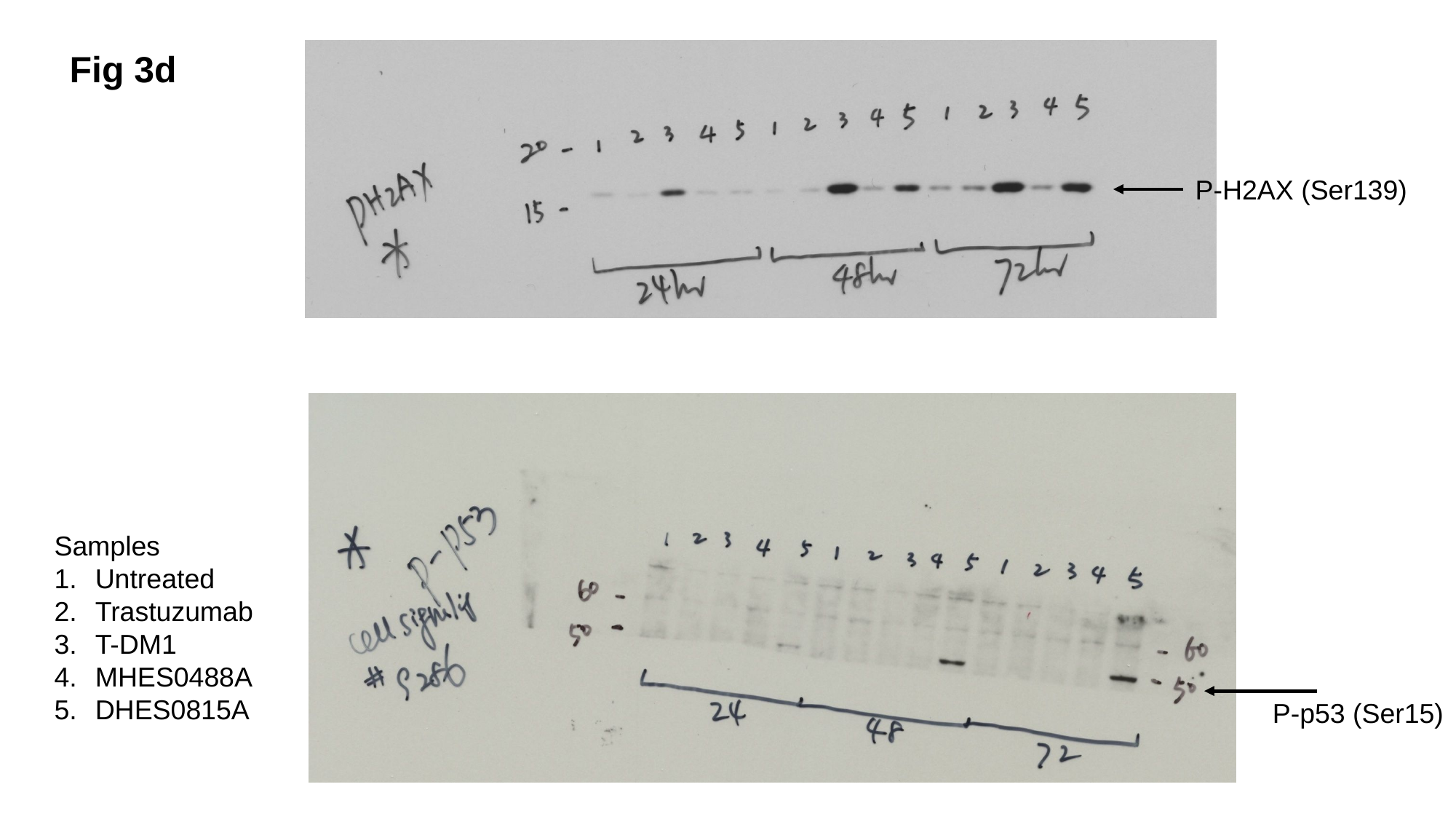

Fig 3d
P-H2AX (Ser139)
Samples
Untreated
Trastuzumab
T-DM1
MHES0488A
DHES0815A
P-p53 (Ser15)

## Slide 5
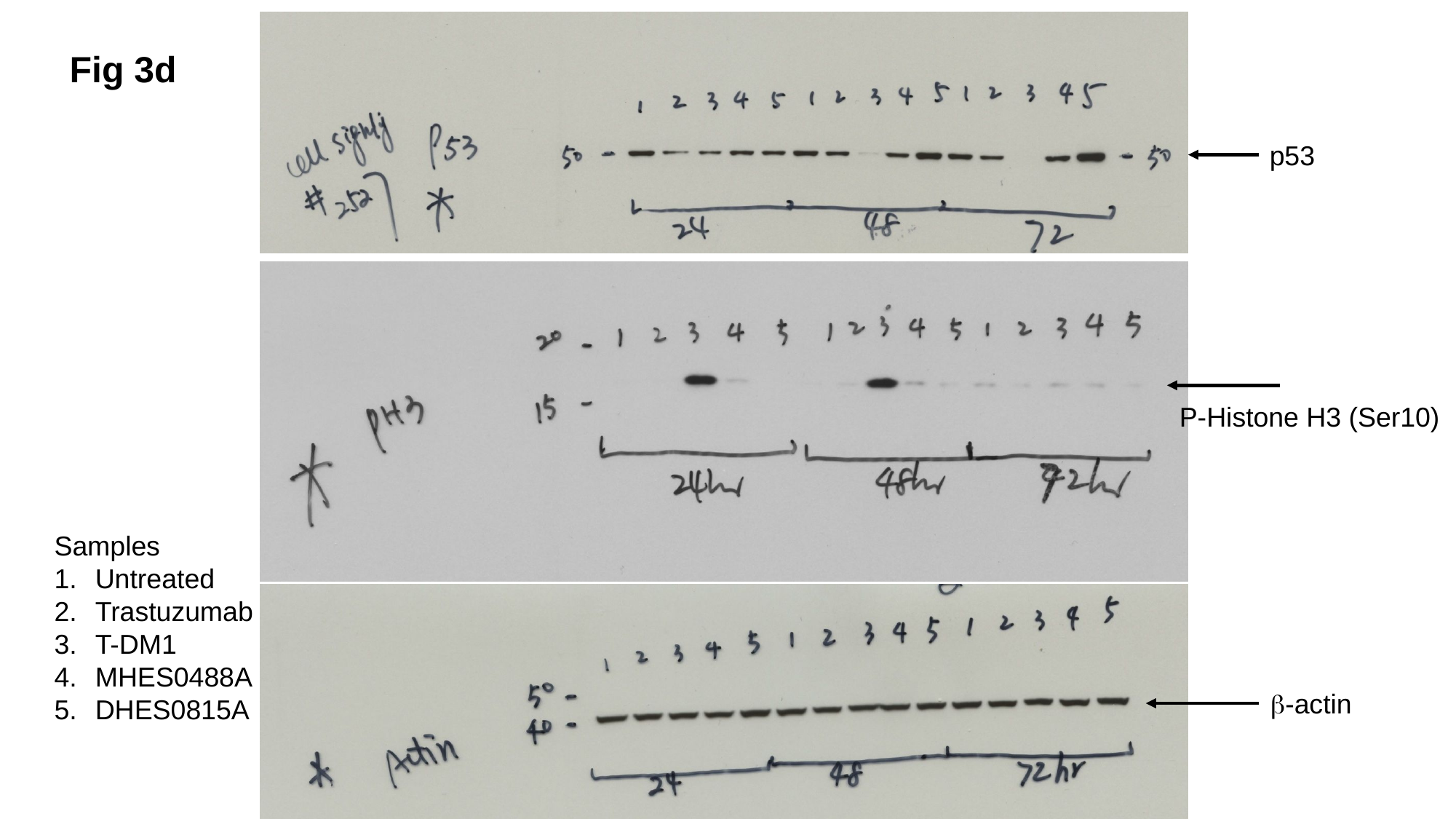

Fig 3d
p53
P-Histone H3 (Ser10)
Samples
Untreated
Trastuzumab
T-DM1
MHES0488A
DHES0815A
b-actin
