# Supplementary material for: The HER2-directed antibody-drug conjugate DHES0815A in advanced and/or metastatic breast cancer: preclinical characterization and phase 1 trial results
Source: Nat Commun. 2024 Jan 11;15:466. doi: 10.1038/s41467-023-44533-z (PMC10784567; doi:10.1038/s41467-023-44533-z)
Supplement: Supplementary file 4 — Source Data [file 41467_2023_44533_MOESM4_ESM.zip › source data files/phase 1/raw data for human PK.pptx]

## Slide 1
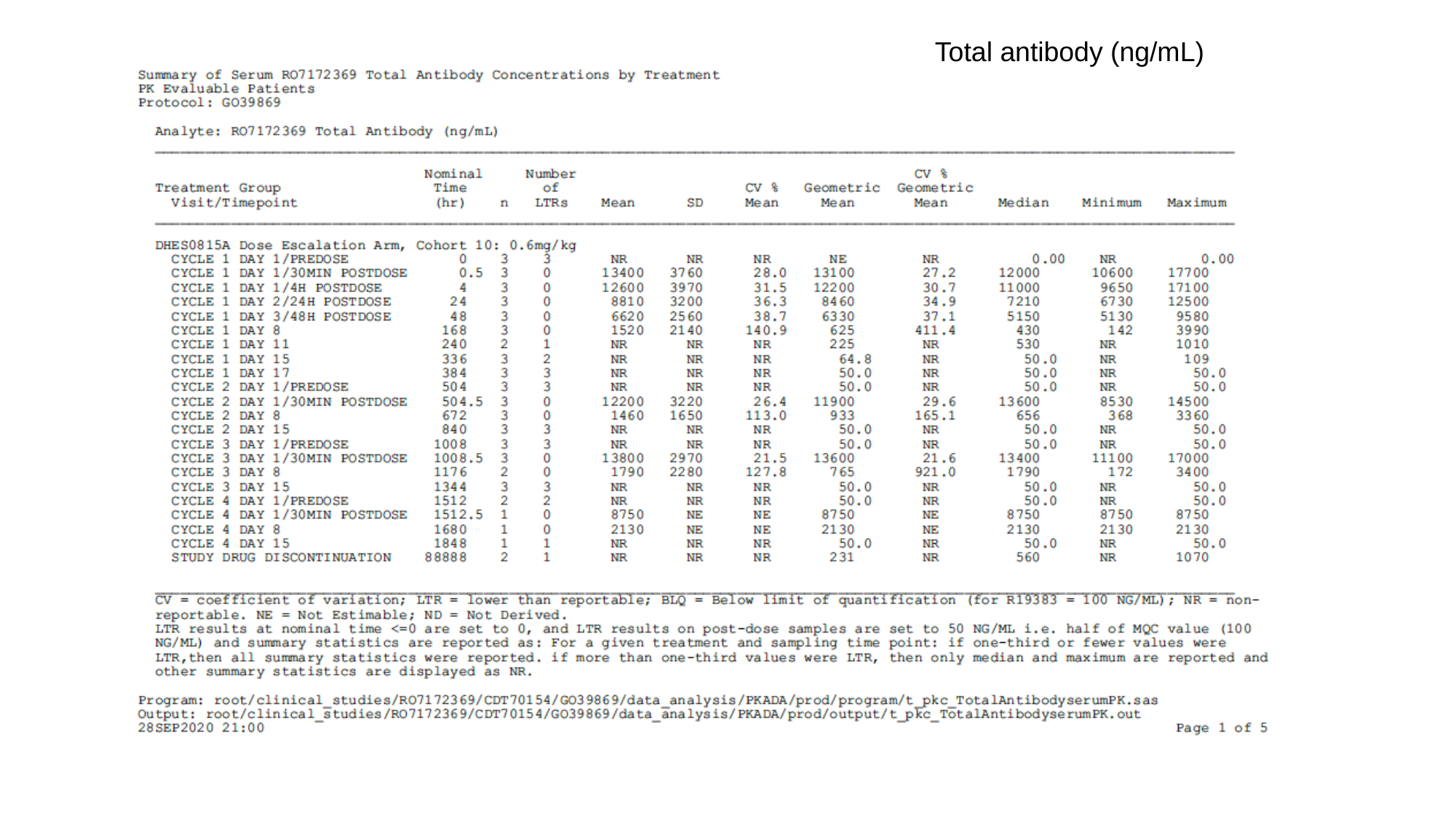

Total antibody (ng/mL)

## Slide 2
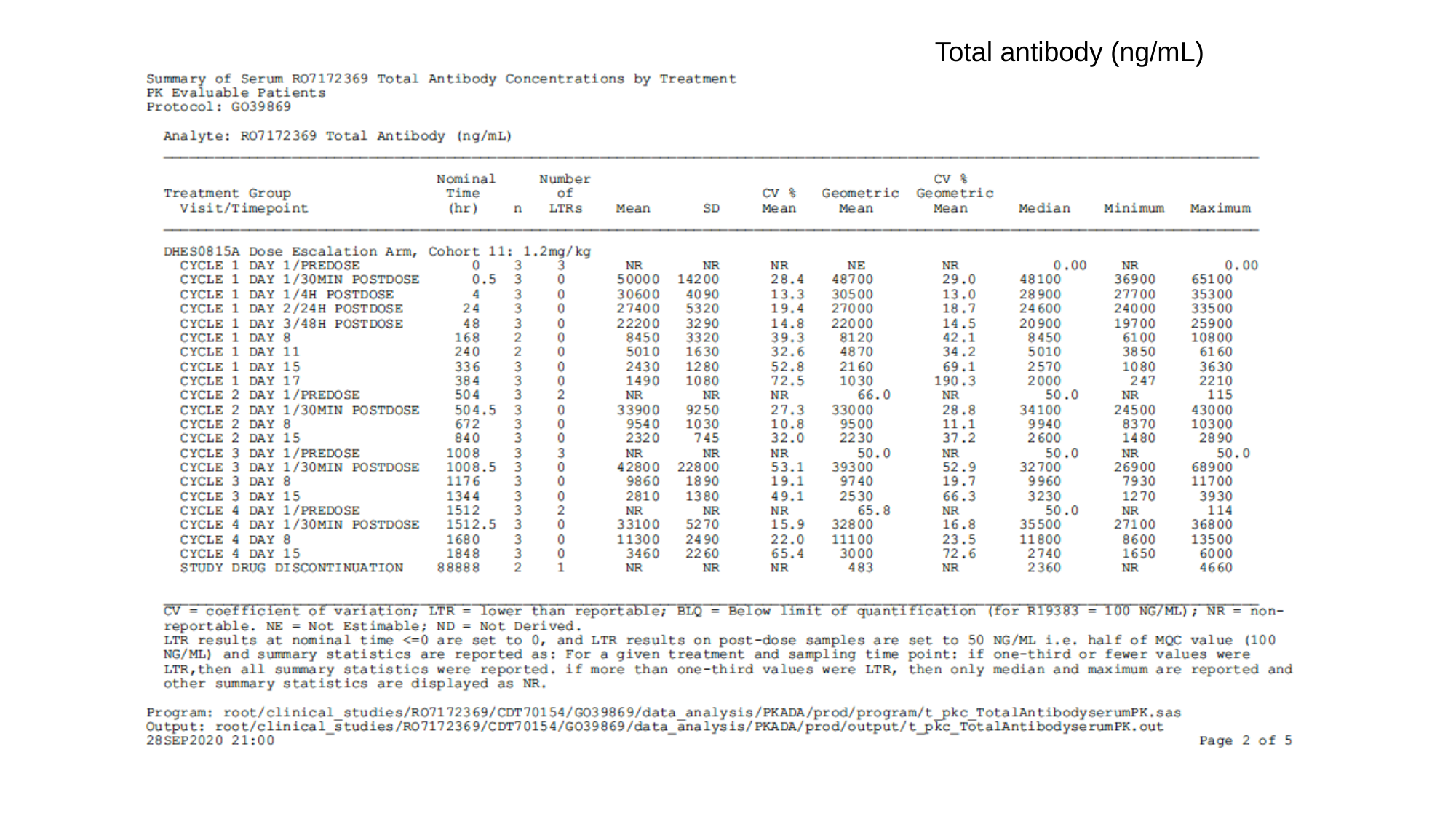

Total antibody (ng/mL)

## Slide 3
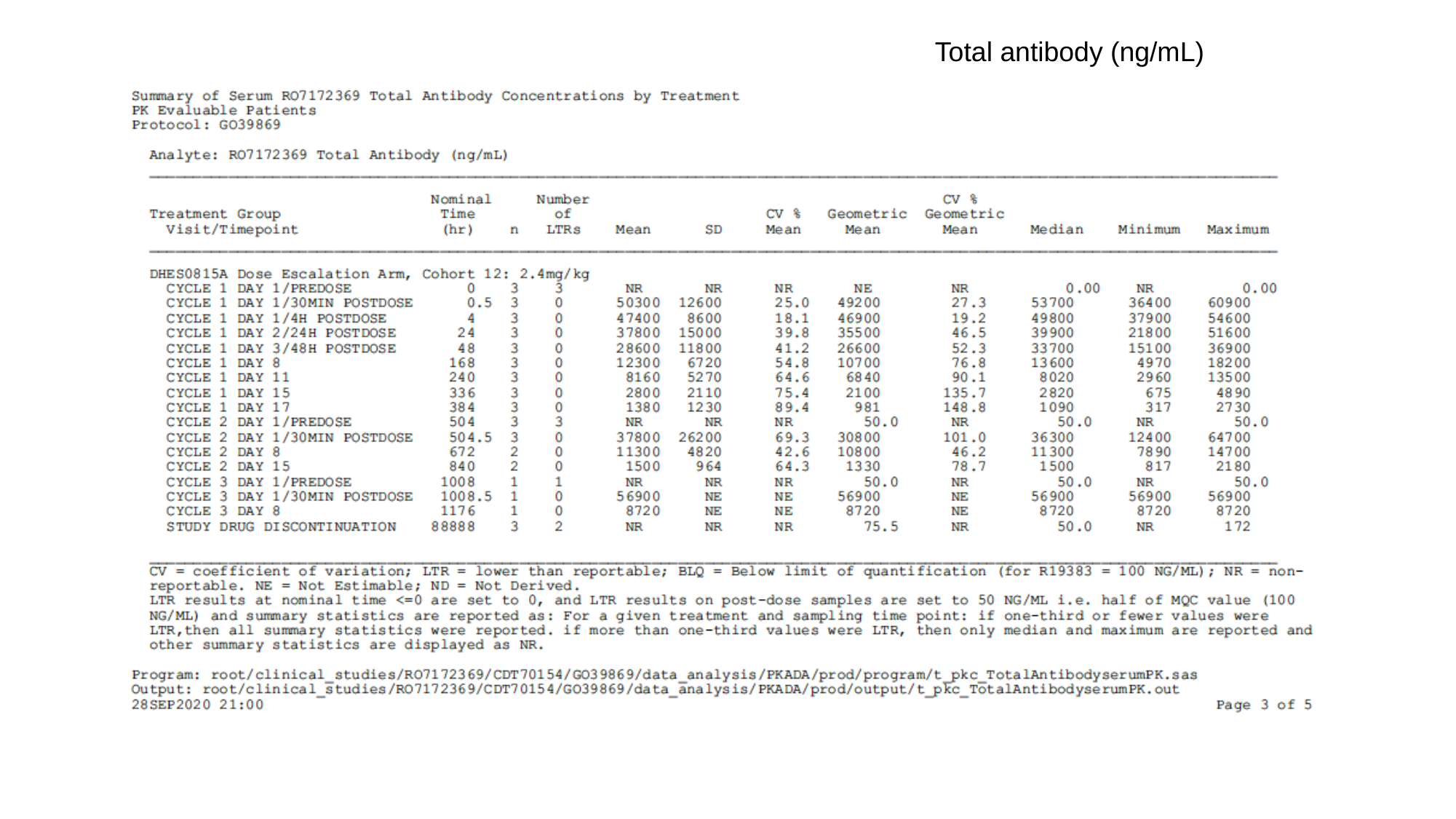

Total antibody (ng/mL)

## Slide 4
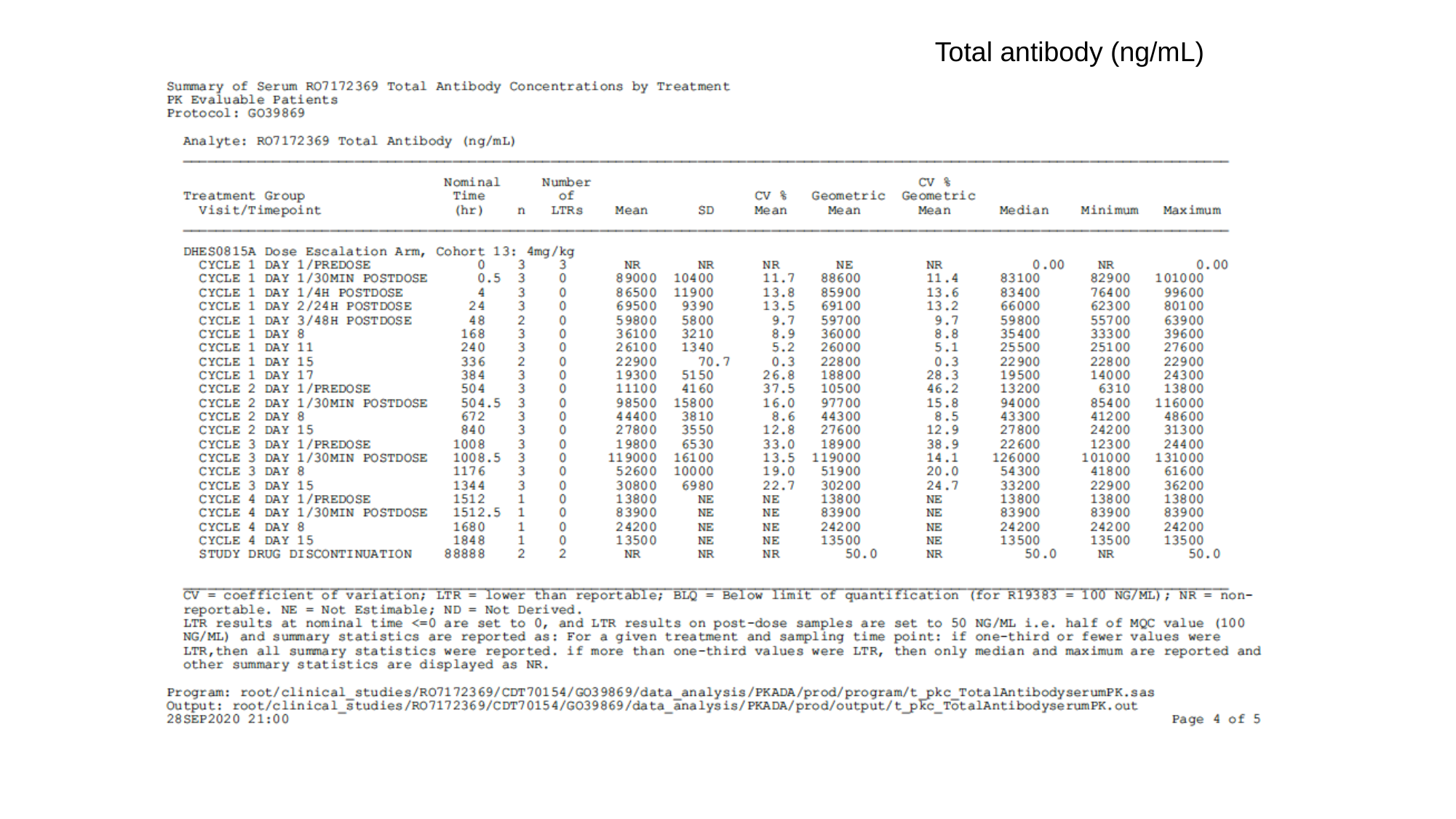

Total antibody (ng/mL)

## Slide 5
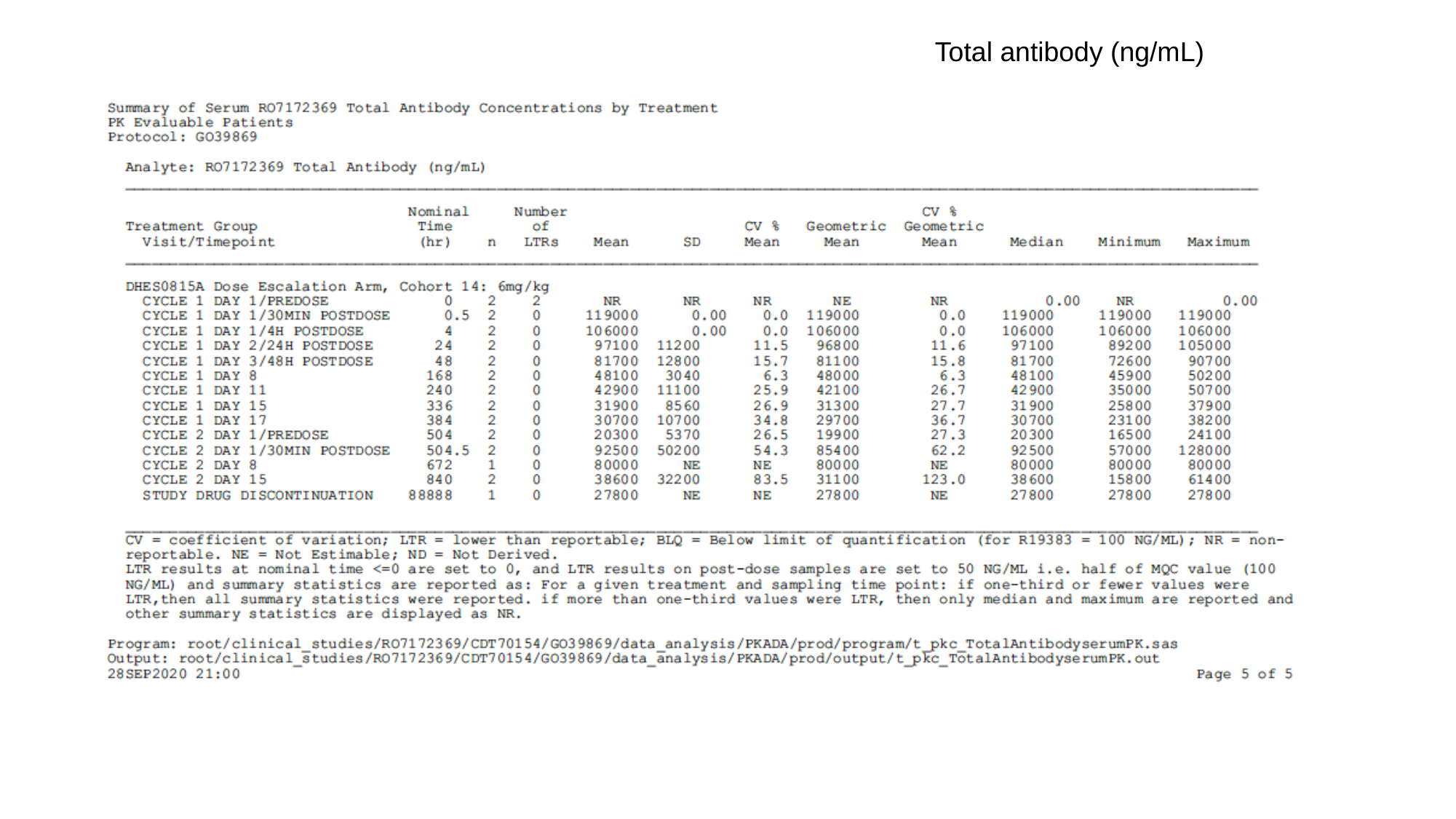

Total antibody (ng/mL)

## Slide 6
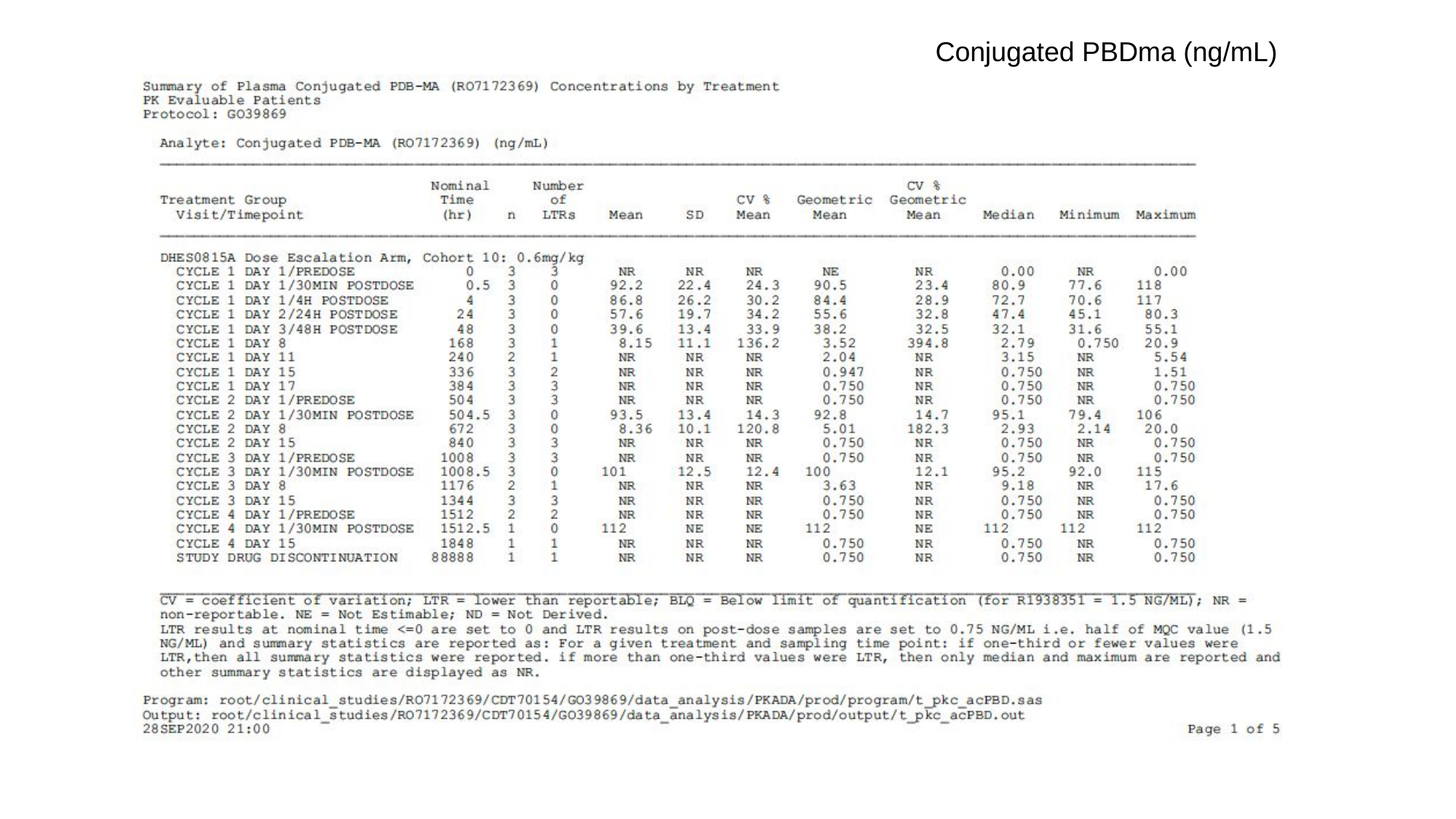

Conjugated PBDma (ng/mL)

## Slide 7
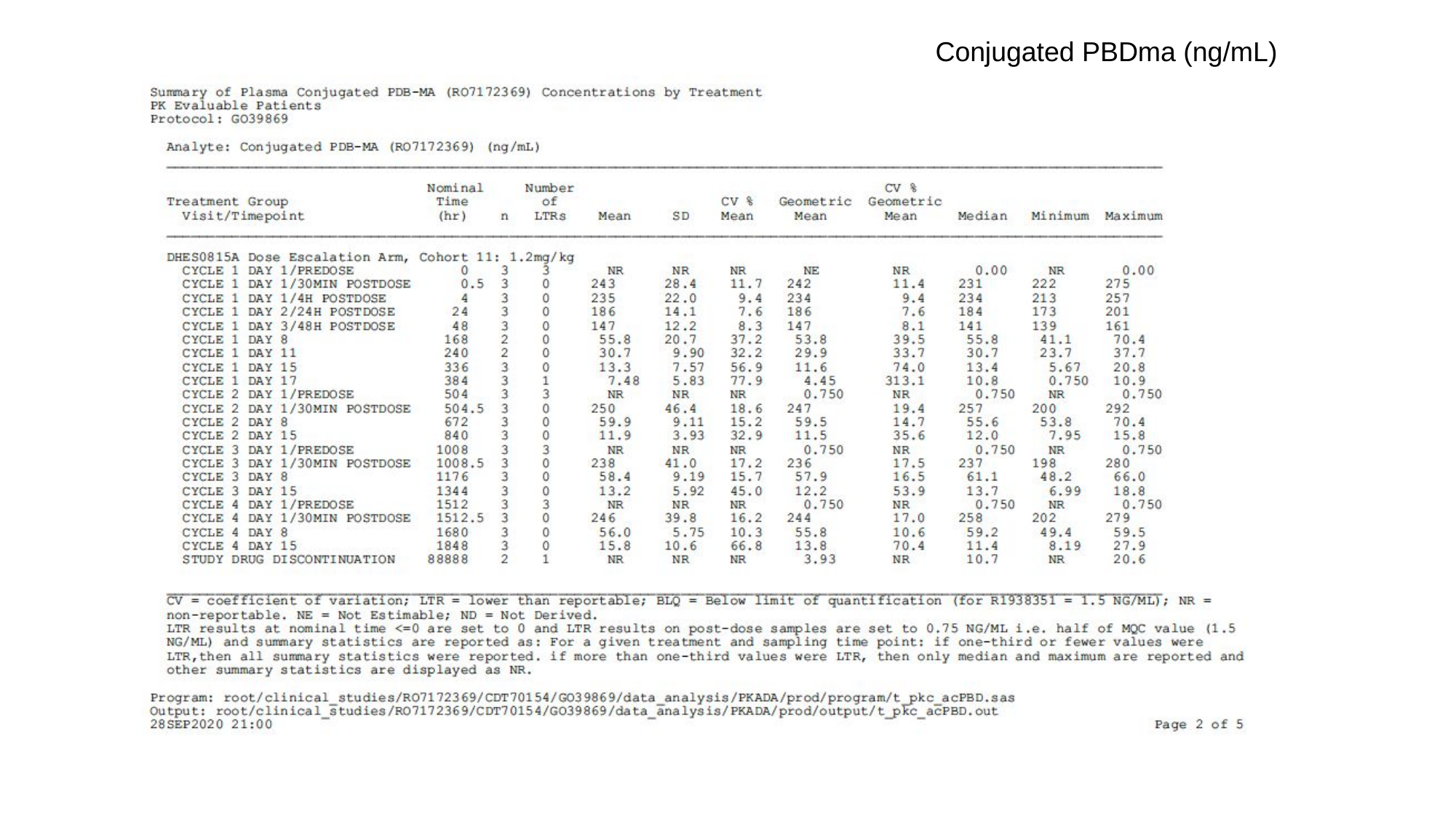

Conjugated PBDma (ng/mL)

## Slide 8
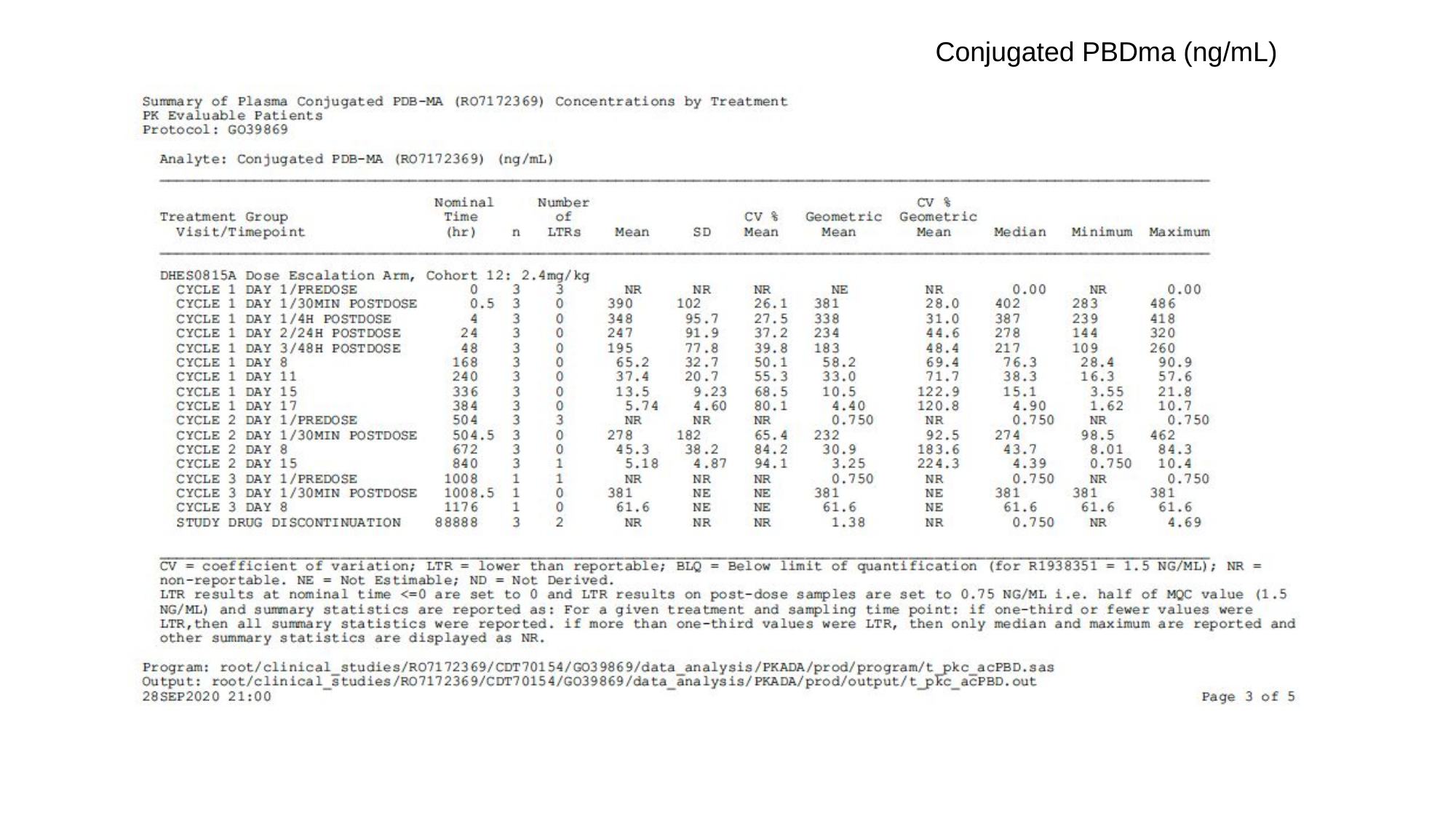

Conjugated PBDma (ng/mL)

## Slide 9
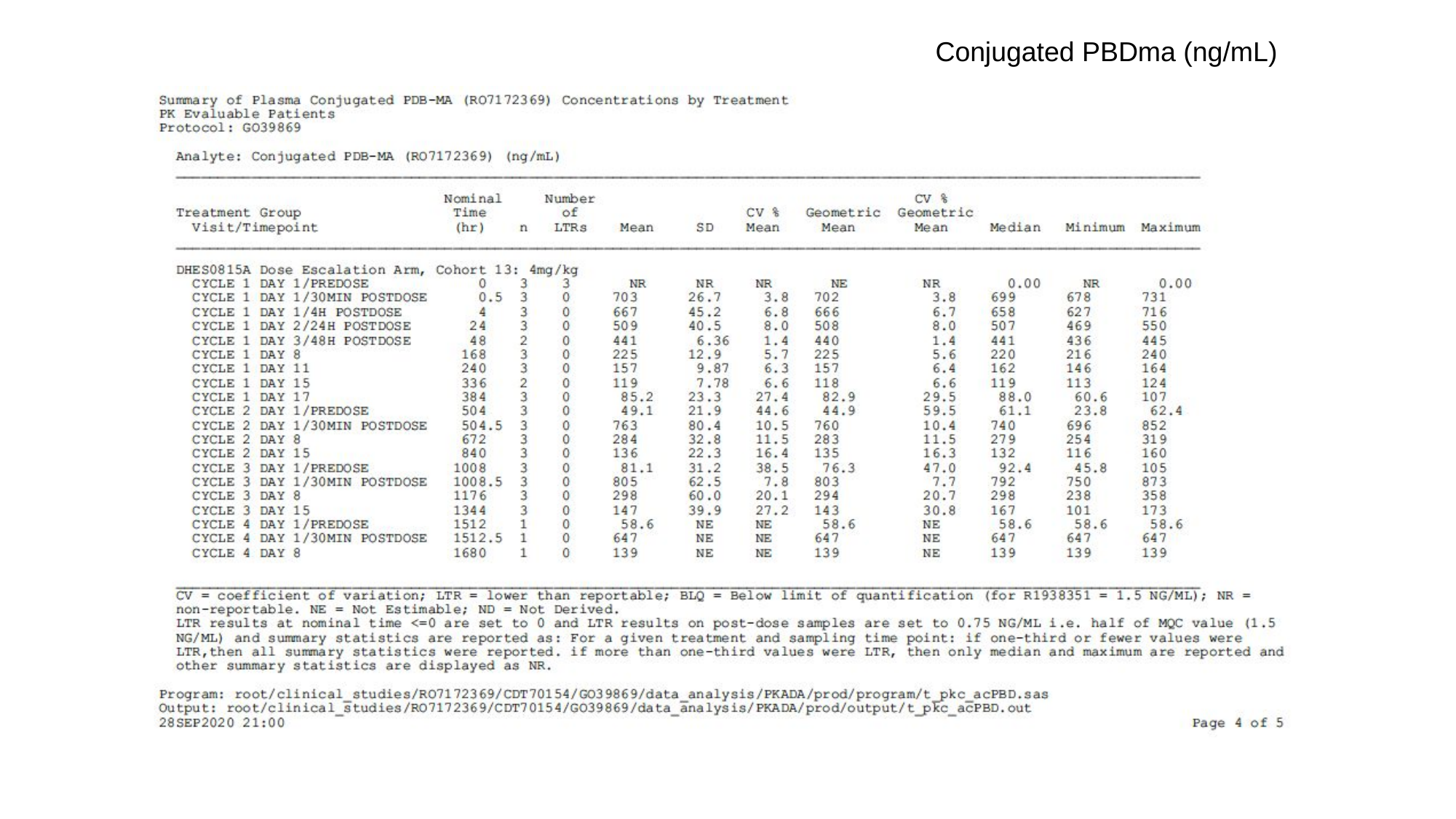

Conjugated PBDma (ng/mL)

## Slide 10
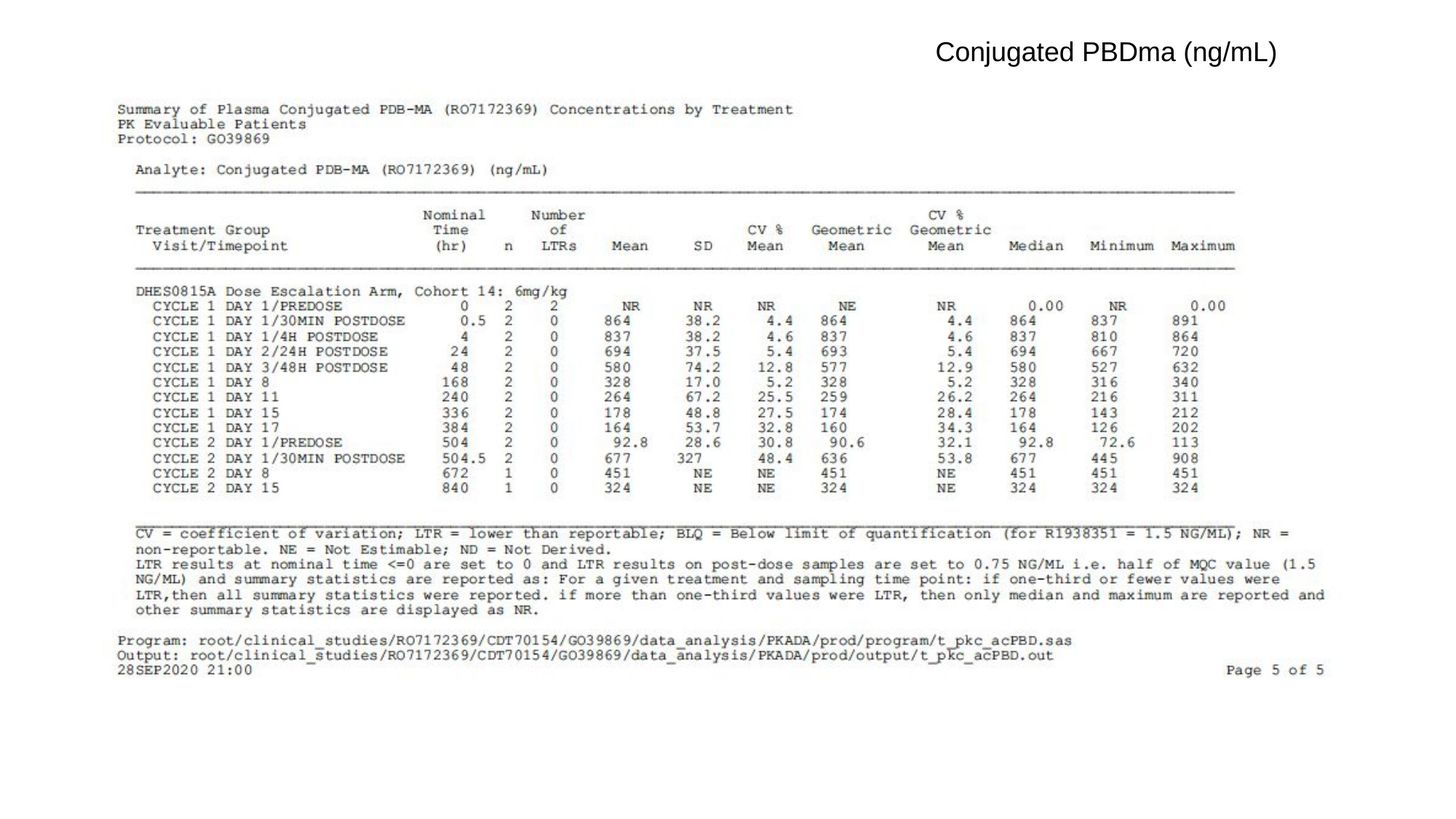

Conjugated PBDma (ng/mL)

## Slide 11
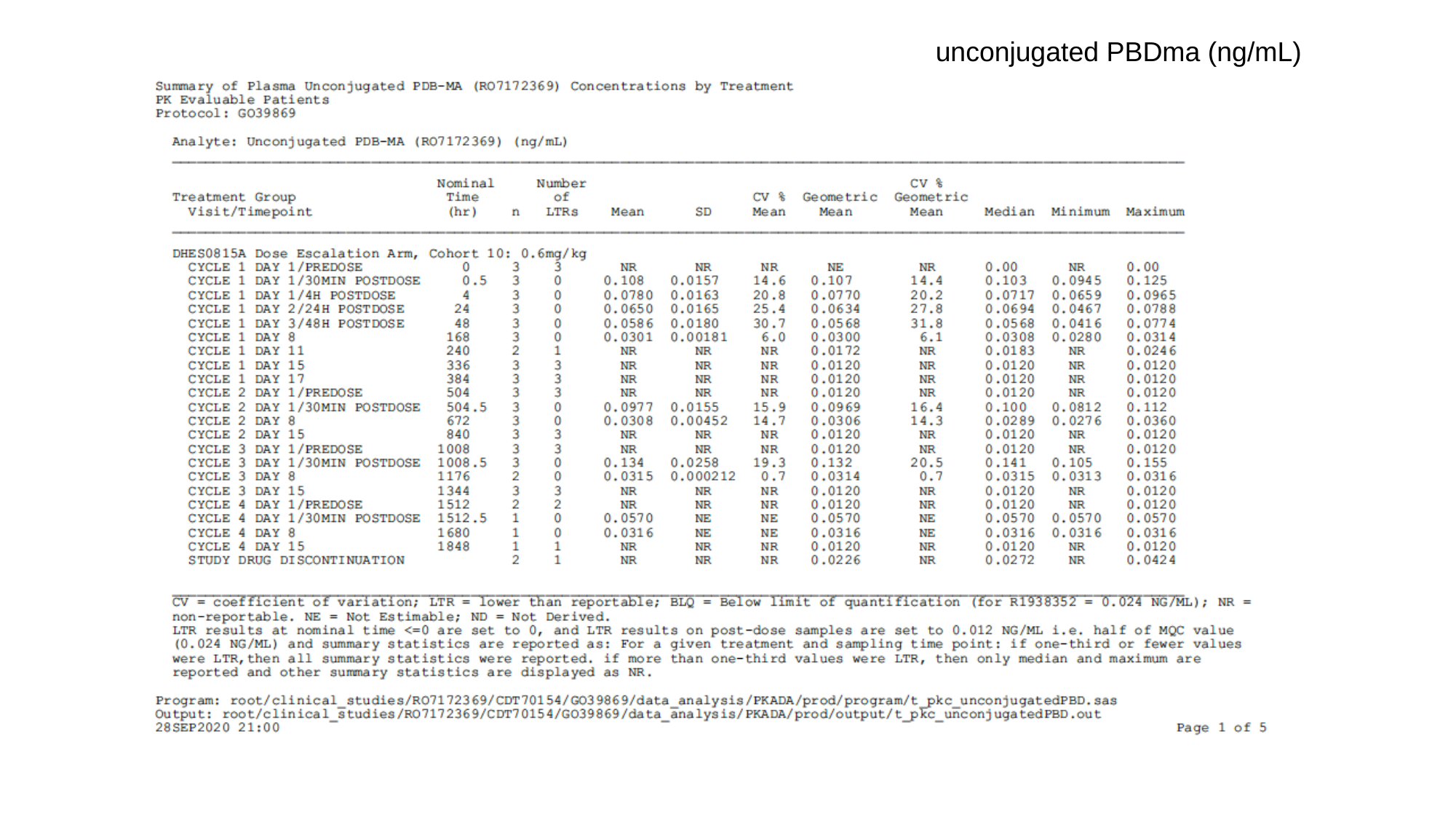

unconjugated PBDma (ng/mL)

## Slide 12
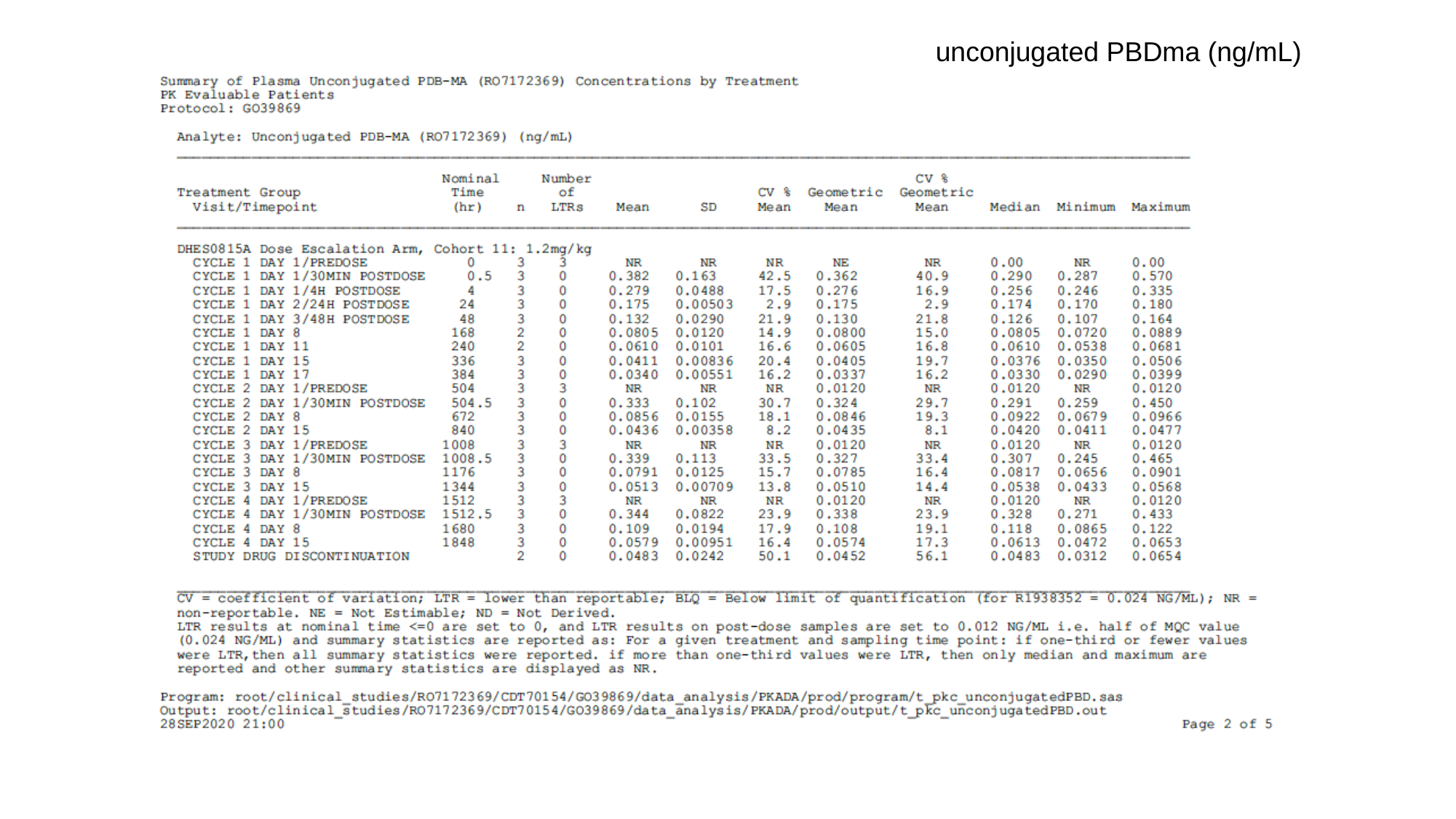

unconjugated PBDma (ng/mL)

## Slide 13
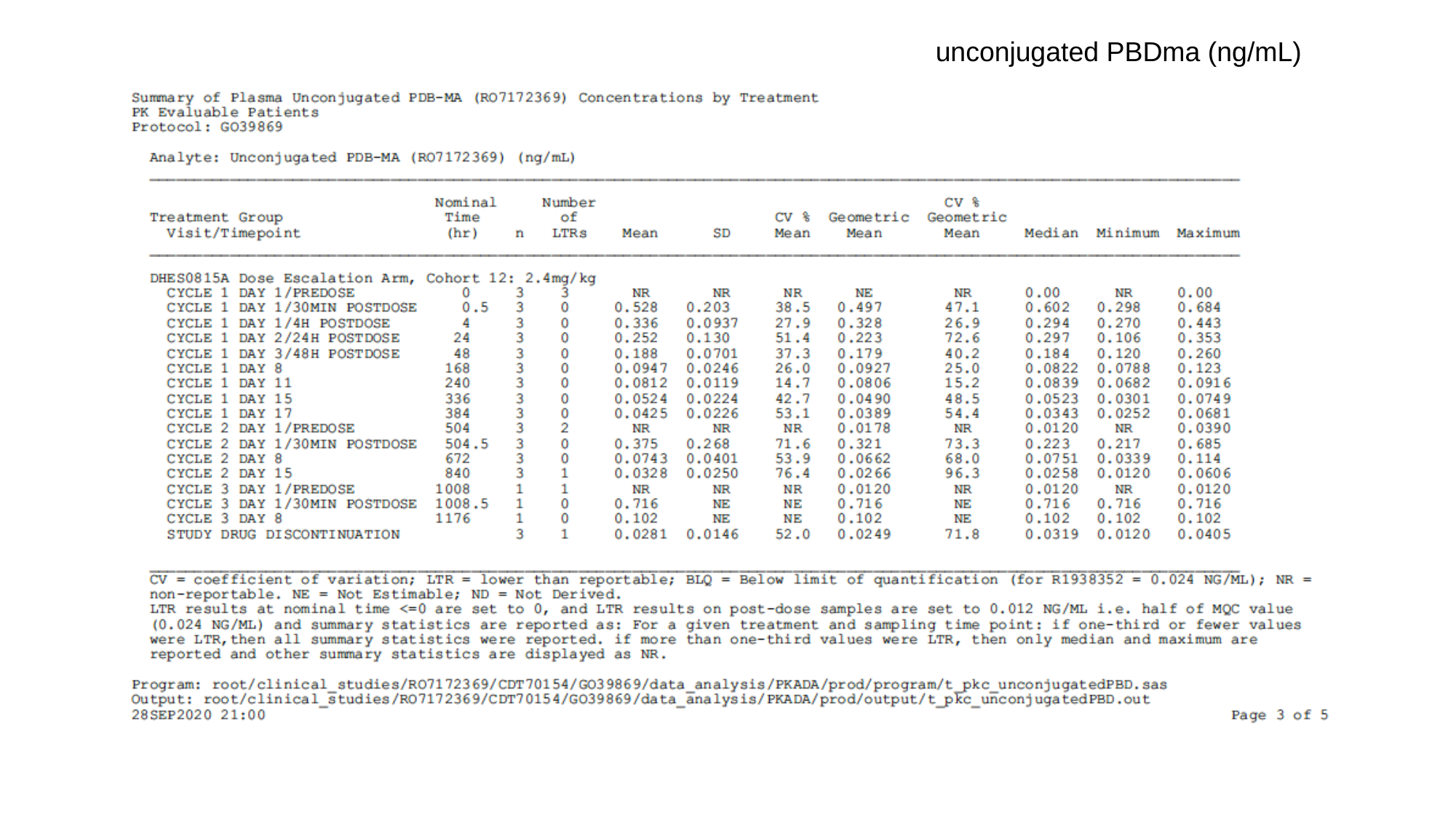

unconjugated PBDma (ng/mL)

## Slide 14
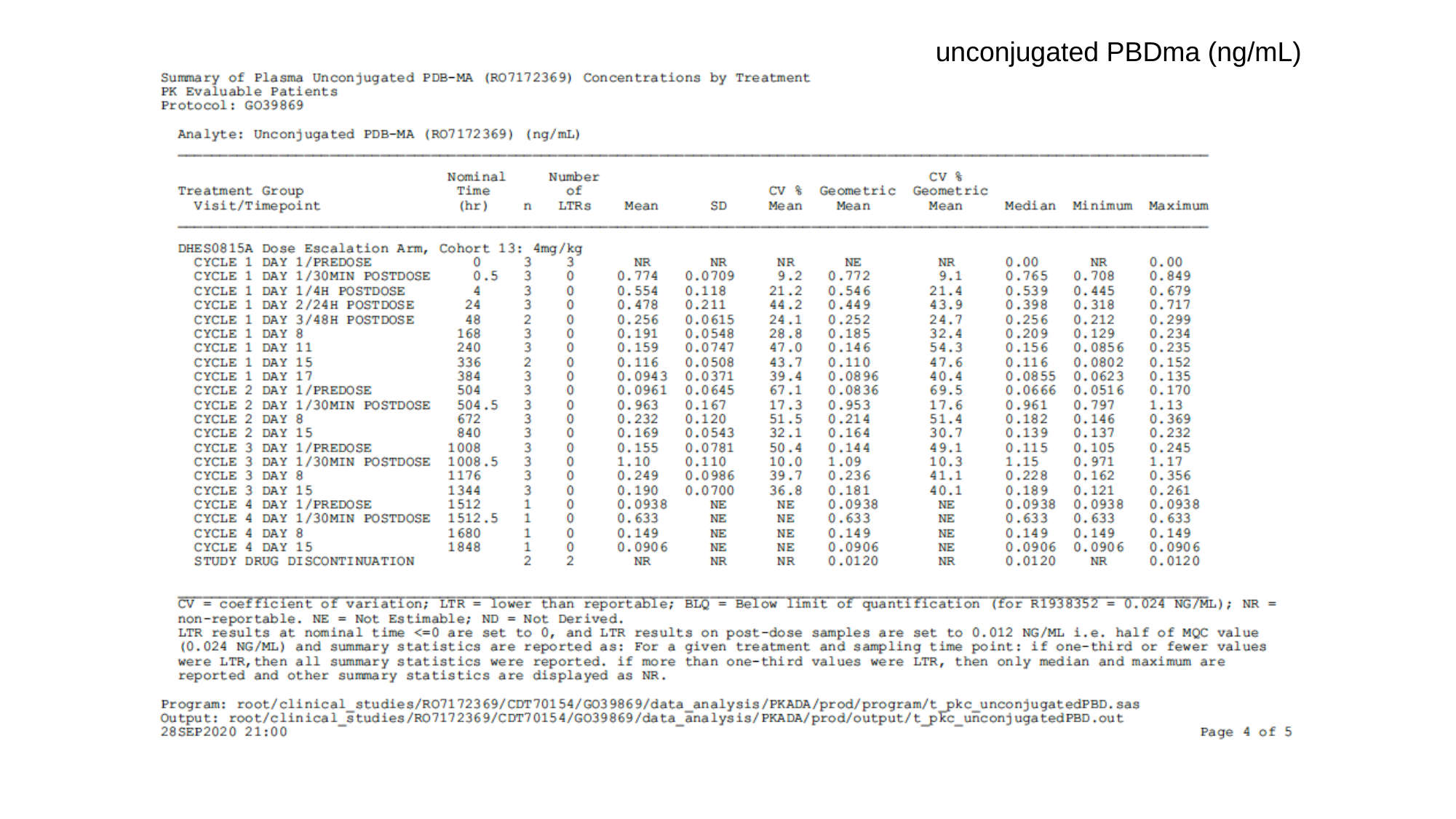

unconjugated PBDma (ng/mL)

## Slide 15
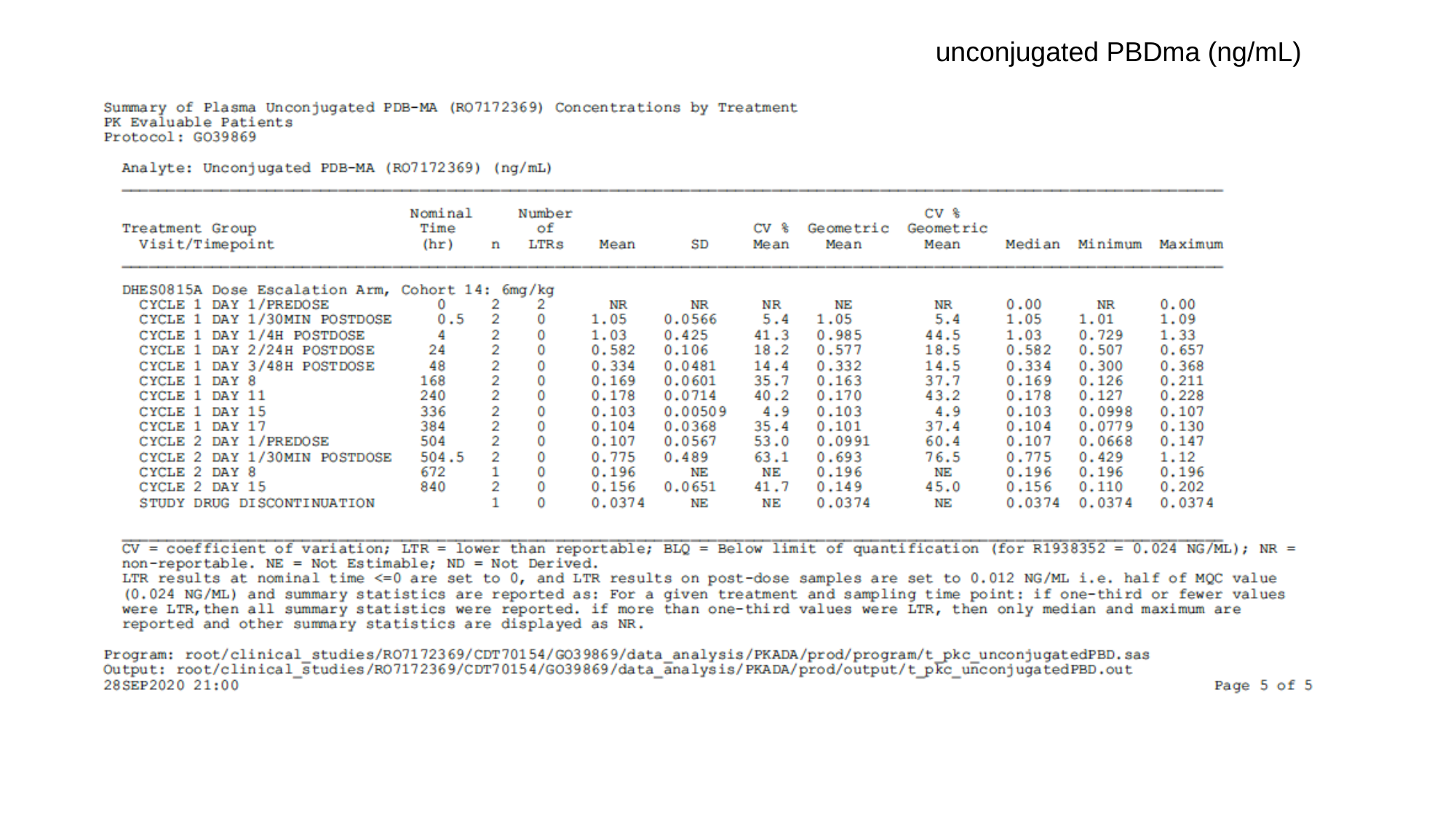

unconjugated PBDma (ng/mL)
