# Supplementary figures and images for: The HER2-directed antibody-drug conjugate DHES0815A in advanced and/or metastatic breast cancer: preclinical characterization and phase 1 trial results
Source: Nat Commun. 2024 Jan 11;15:466. doi: 10.1038/s41467-023-44533-z (PMC10784567; doi:10.1038/s41467-023-44533-z)

## Slide 1
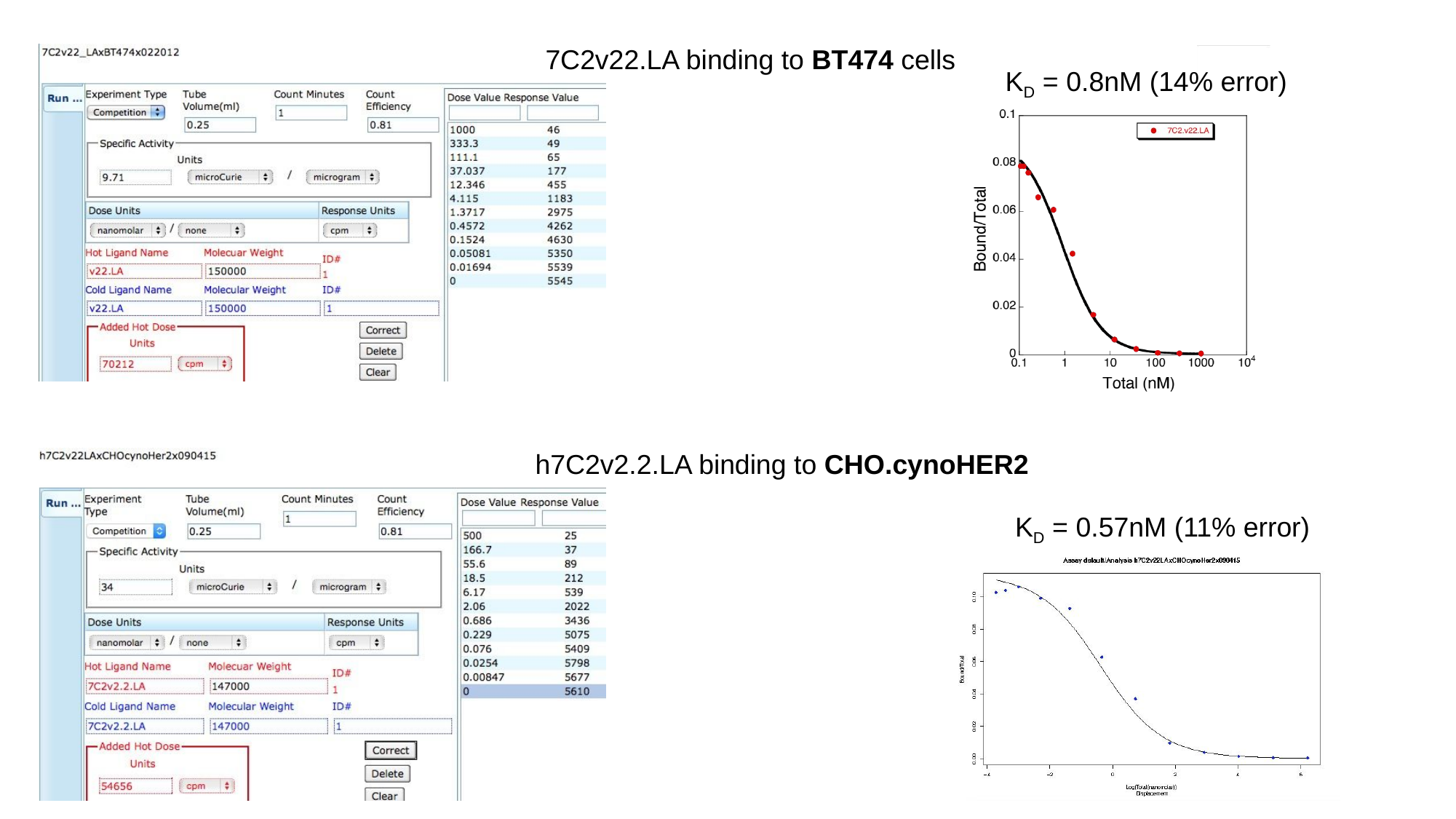

7C2v22.LA binding to BT474 cells
KD = 0.8nM (14% error)
h7C2v2.2.LA binding to CHO.cynoHER2
KD = 0.57nM (11% error)

Supplement: Supplementary file 4 — Source Data [file 41467_2023_44533_MOESM4_ESM.zip › source data files/supp figures/Supp fig 1 binding.pptx]
